# Supplementary material for: Using Genetics to Assess the Role of Acetate in Ischemic Heart Disease, Diabetes, and Sex-Hormone-Related Cancers: A Mendelian Randomization Study
Source: Nutrients. 2024 Oct 29;16(21):3674. doi: 10.3390/nu16213674 (PMC11547320; doi:10.3390/nu16213674)
Supplement: Supplementary file 1 [file nutrients-16-03674-s001.zip › Supplemental Figures.pdf]

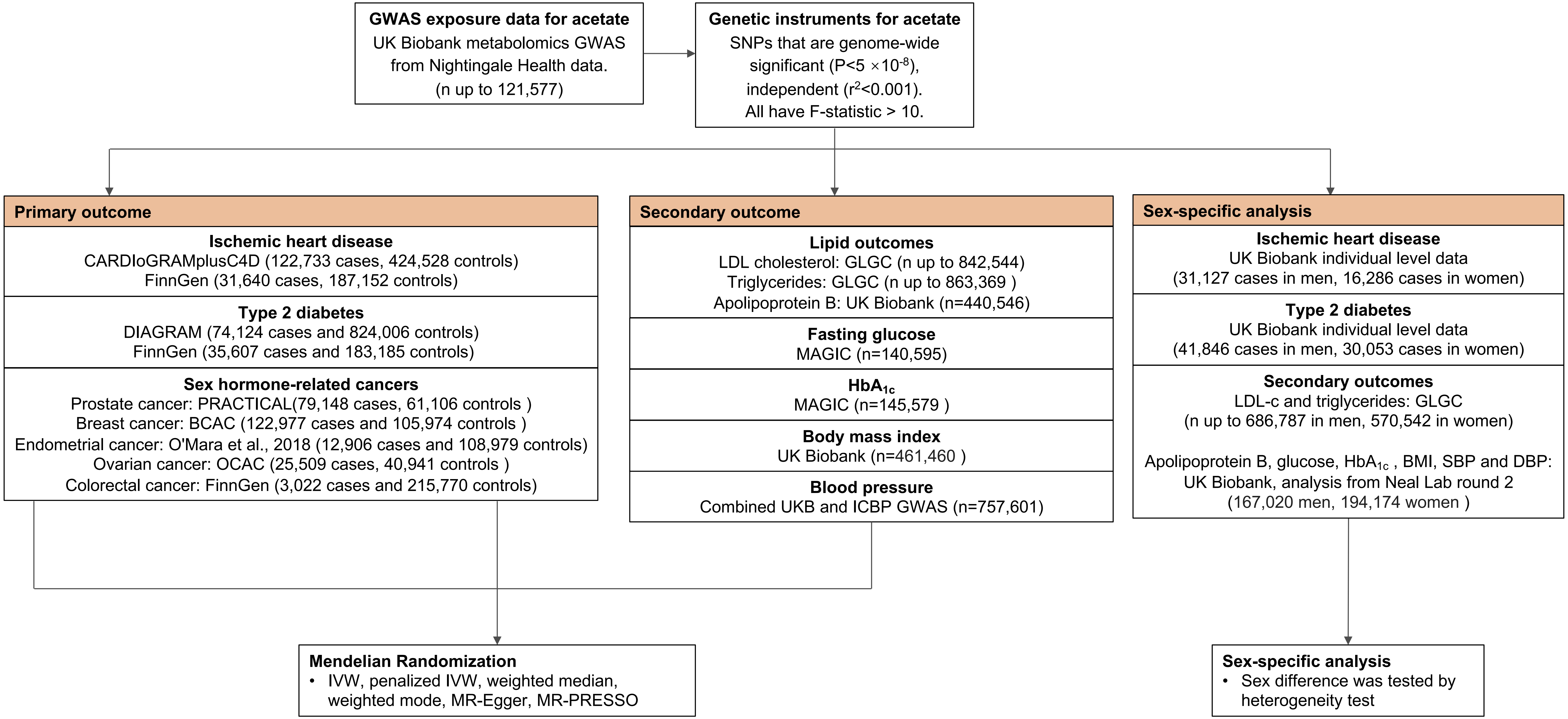

Figure S1: Flow diagram of study design

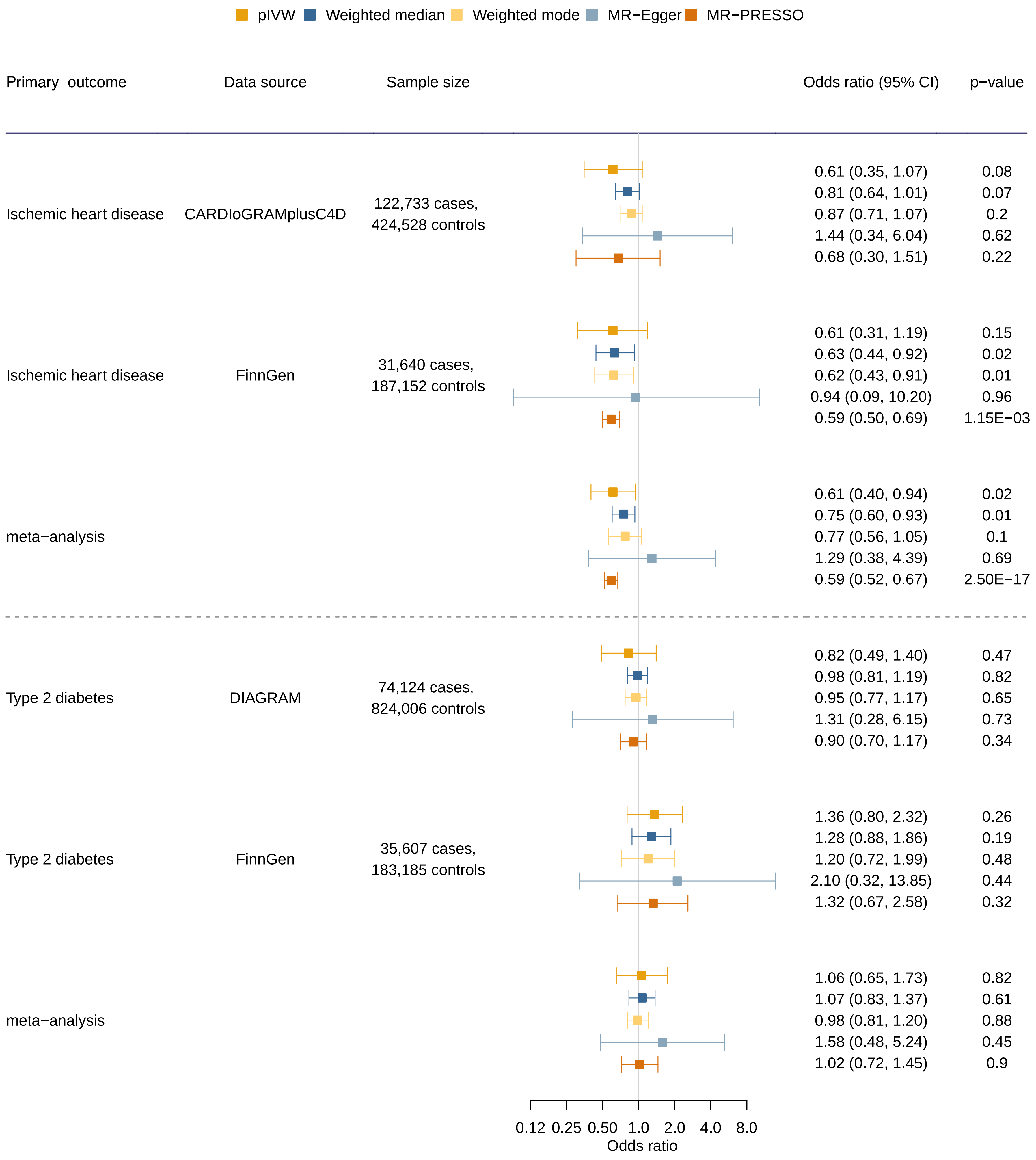

Figure S2: Sensitivity analysis on the overall association of genetically predicted acetate with ischemic heart disease and type 2 diabetes using different analytic methods

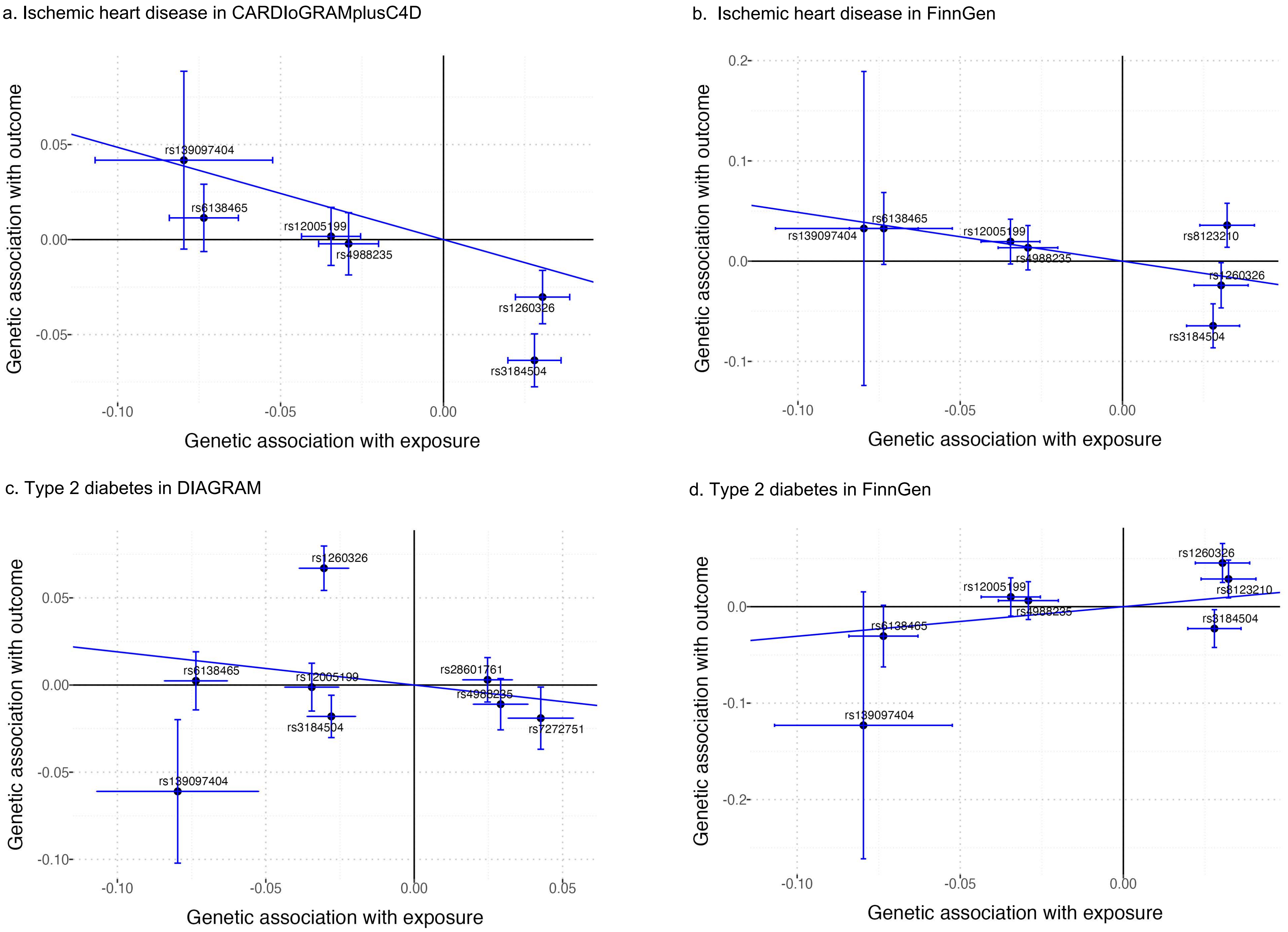

Figure S3: Scatter plot showing the overall association of each SNP with acetate and with ischemic heart disease in CARDIoGRAMplusC4D (a) and FinnGen (b), with type 2 diabetes in Diagram (c), and FinnGen (d)

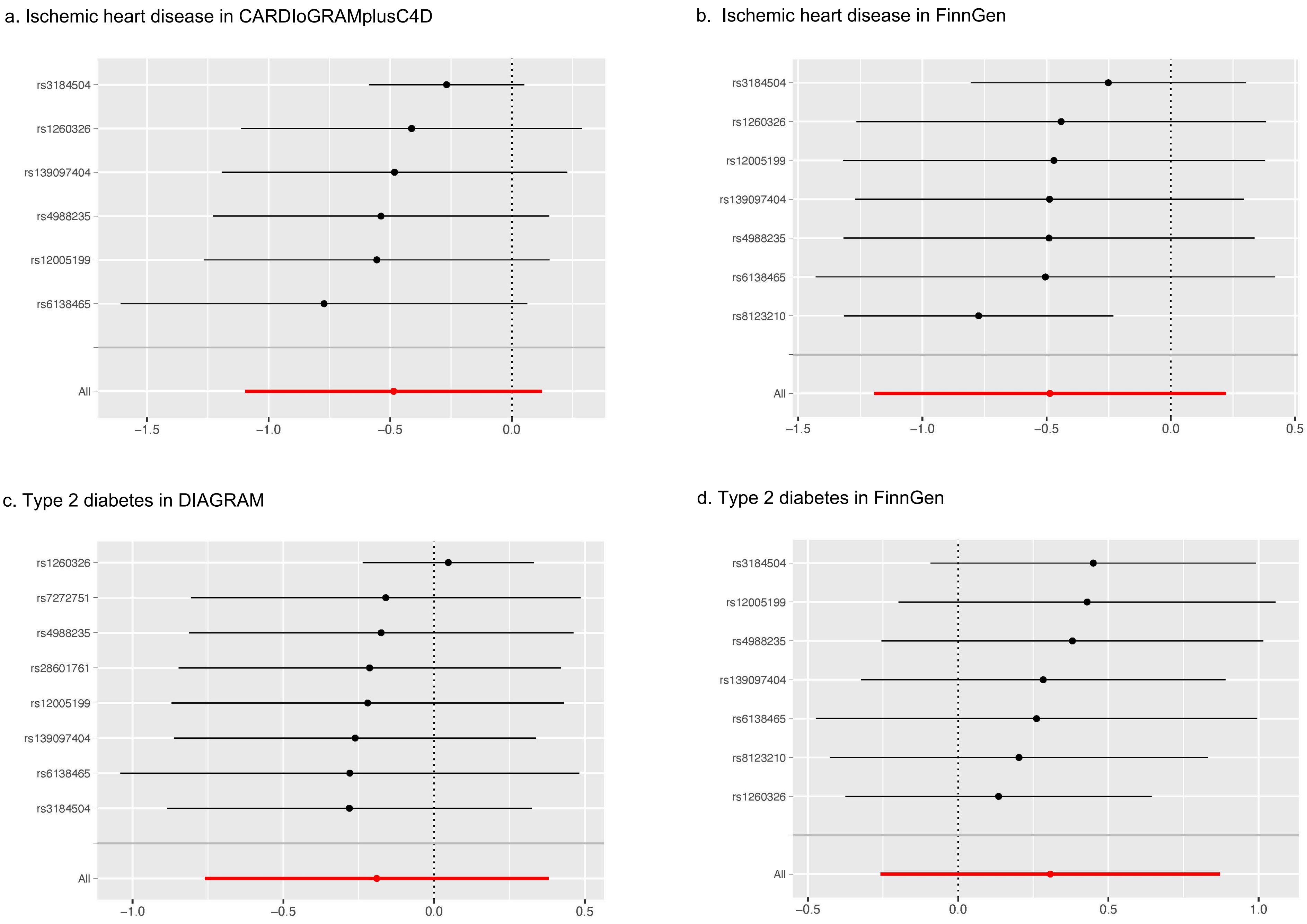

Figure S4: Leave−one−out sensitivity analysis on the overall association of genetically predicted acetate with ischemic heart disease in CARDIoGRAMplusC4D (a) and FinnGen (b), with type 2 diabetes in Diagram (c), and FinnGen (d)

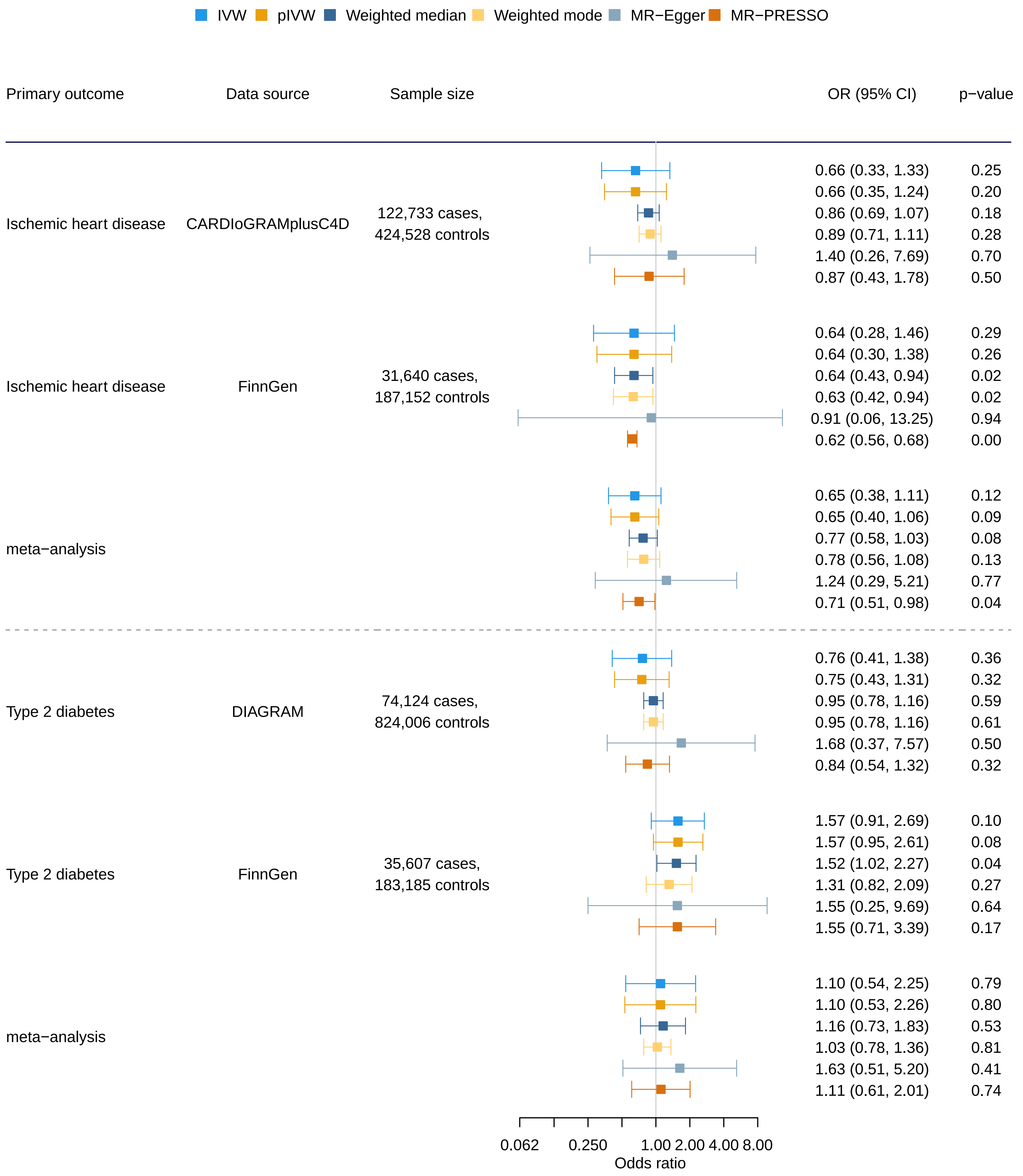

Figure S5: Sensitivity analysis on the overall association of genetically predicted acetate with ischemic heart disease (removing rs1260326) and type 2 diabetes (removing rs3184504) using different analytic methods

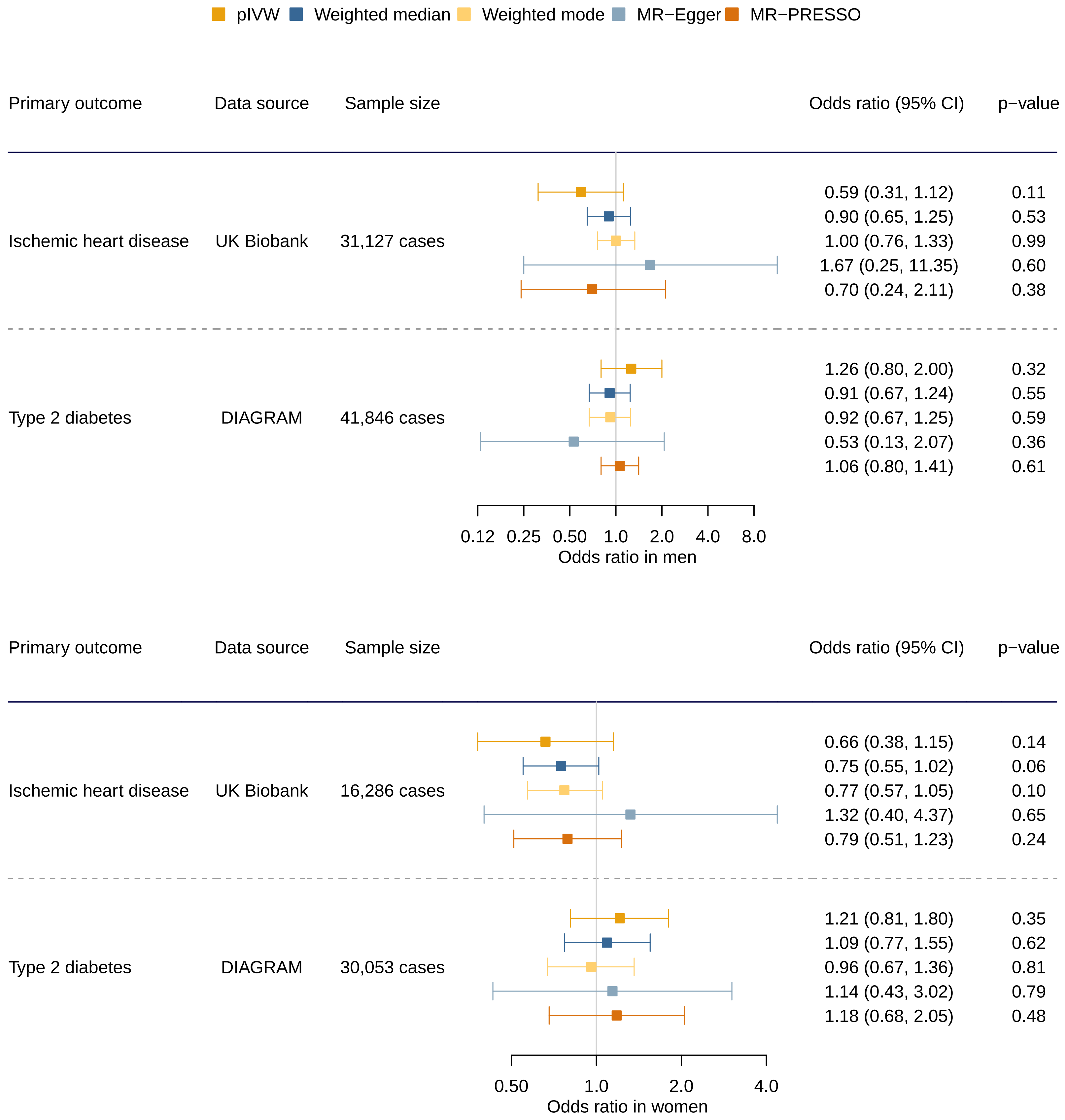

Figure S6: Sensitivity analysis on the sex-specific association of genetically predicted acetate with ischemic heart disease and type 2 diabetes using different analytic methods

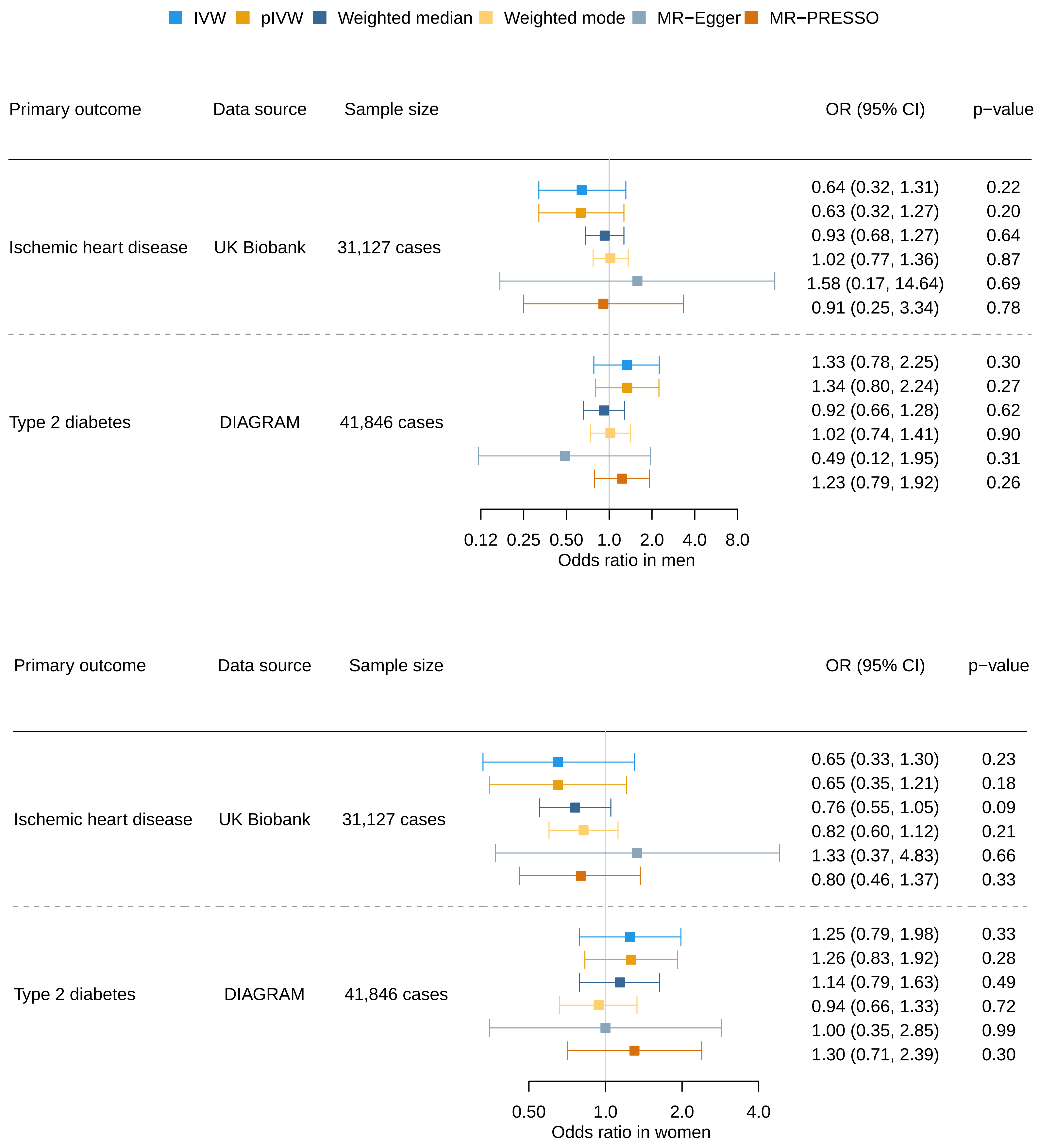

Figure S7: Sensitivity analysis on the sex-specific association of genetically predicted acetate with ischemic heart disease (removing rs1260326) and type 2 diabetes (removing rs3184504) using different analytic methods

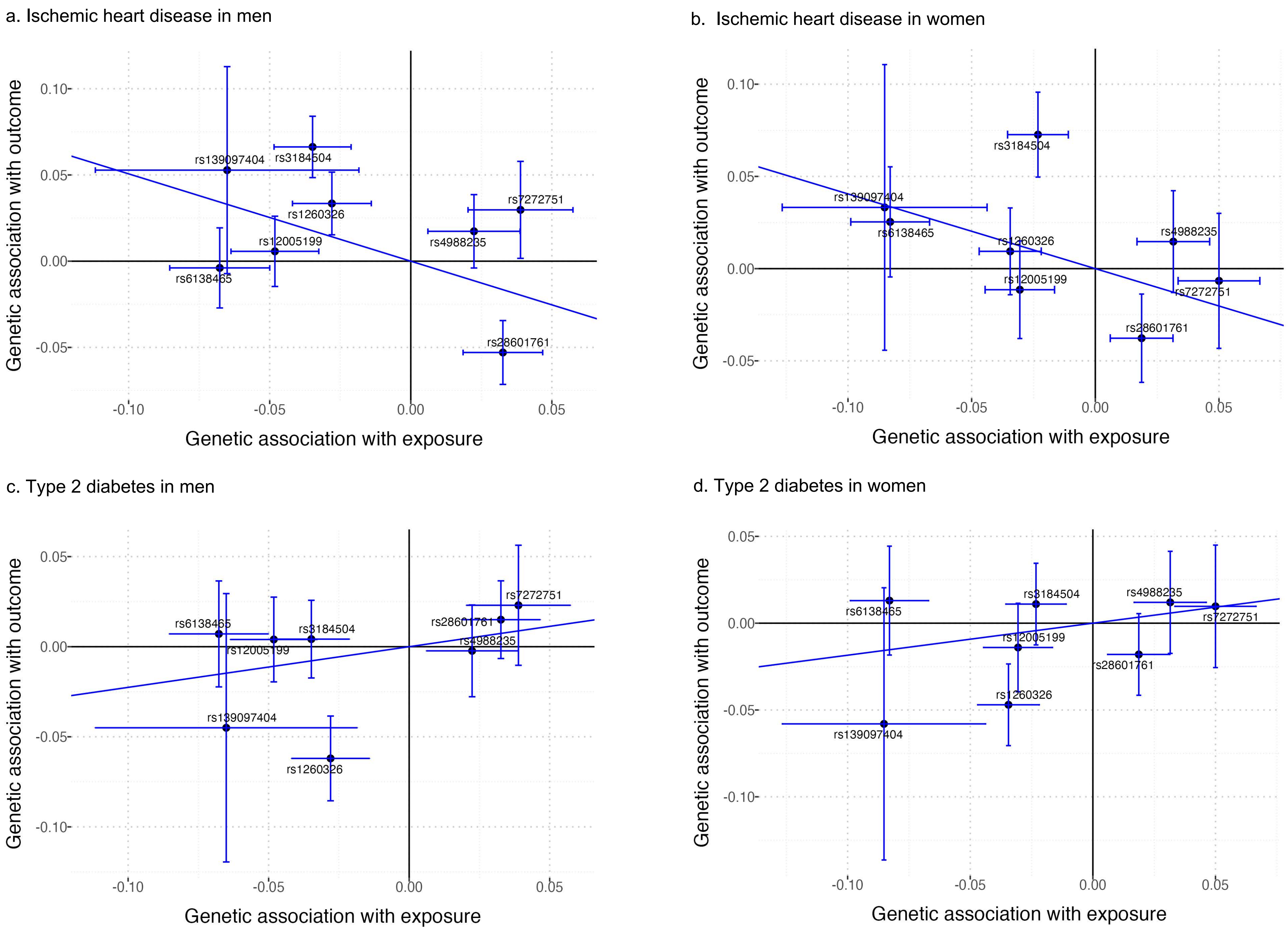

Figure S8: Scatter plot showing the sex-specific association of each SNP with acetate and with ischemic heart disease in men (a), and women (b), with type 2 diabetes in men (c), and women (d)

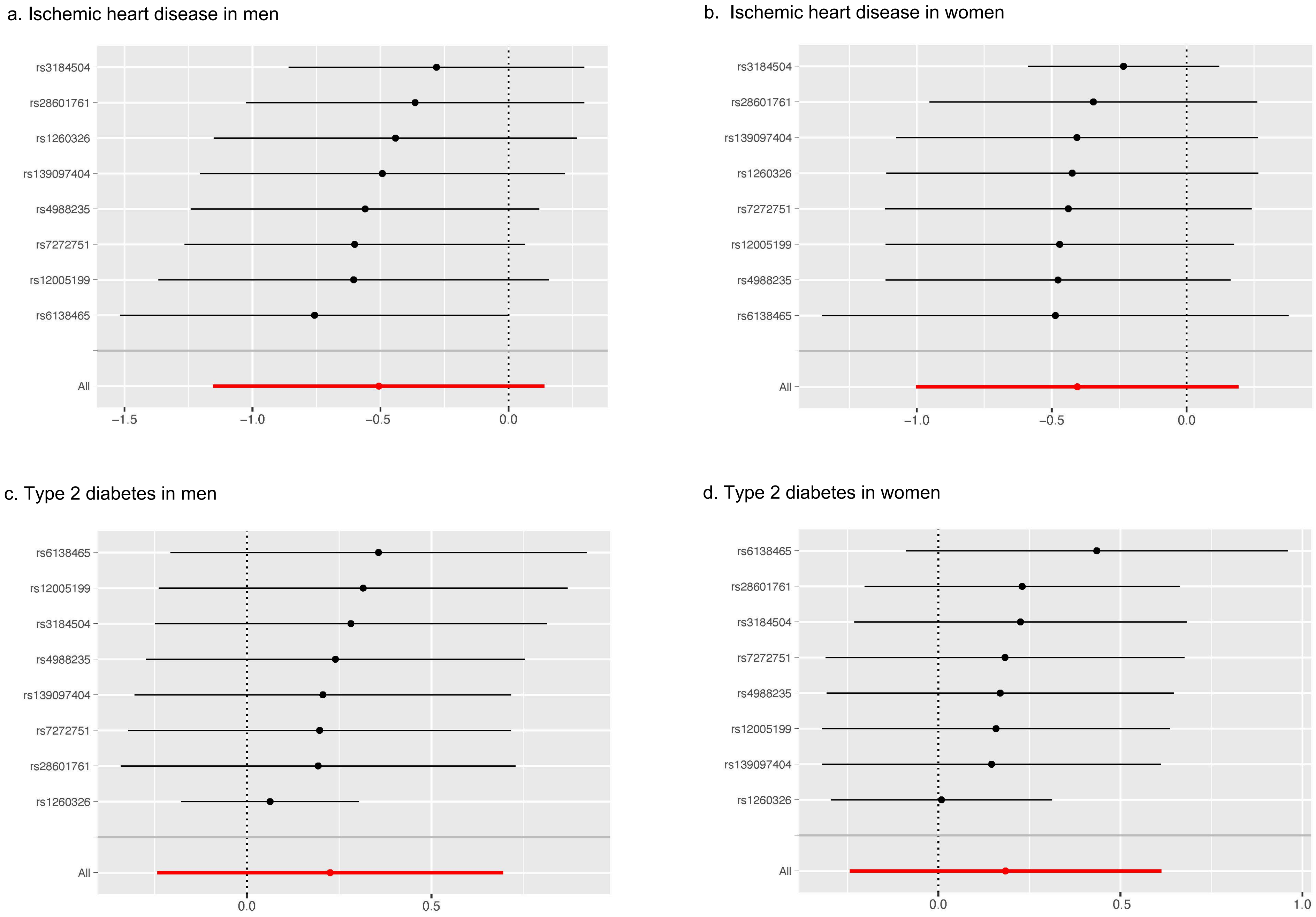

Figure S9: Leave−one−out sensitivity analysis on the sex-specific association of genetically predicted acetate with ischemic heart disease in men (a), and women (b), with type 2 diabetes in men (c), and women (d)

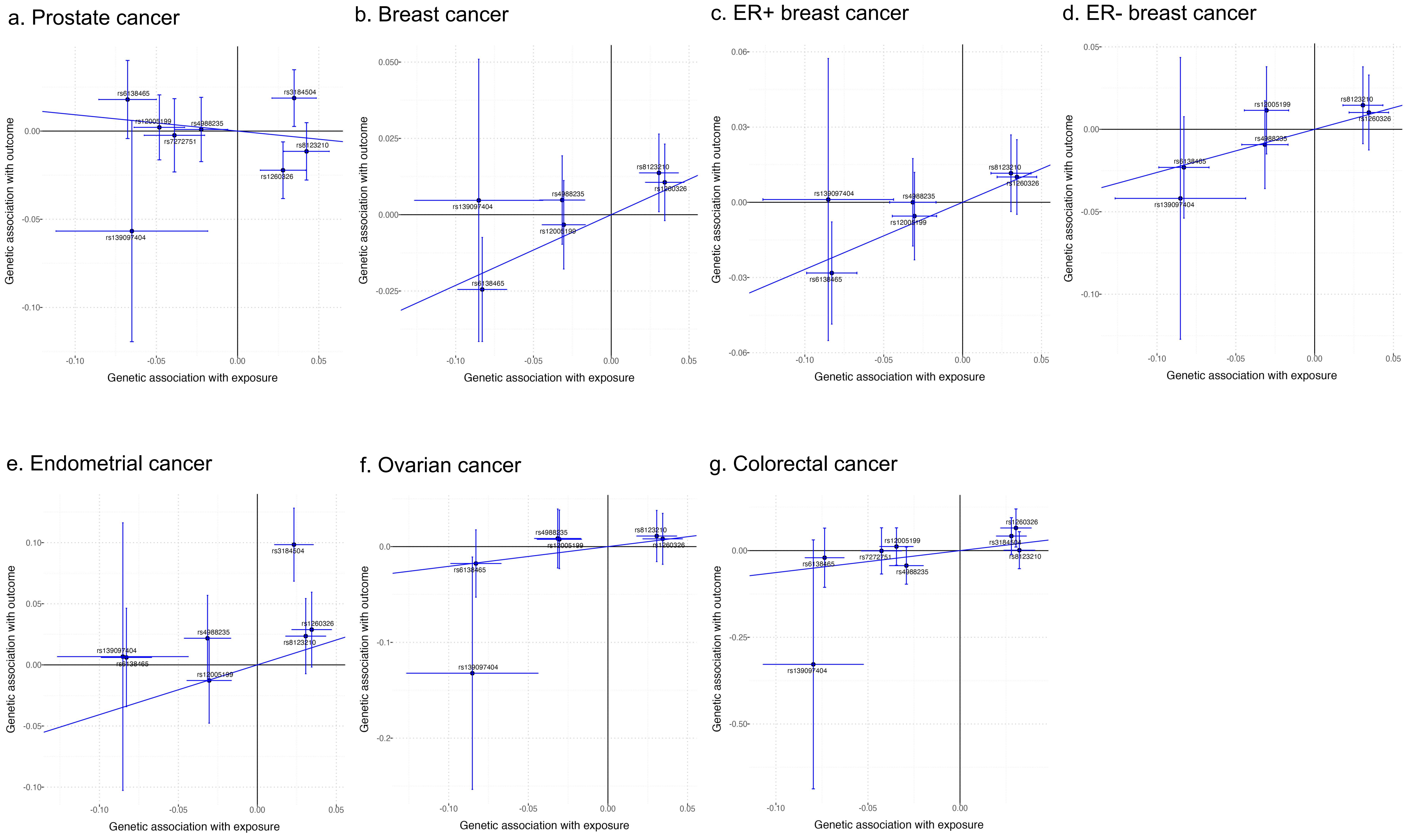

Figure S10: Scatter plot showing the association of each SNP with acetate and with prostate cancer (a), breast cancer (b), ER+ breast cancer (c), ER− breast cancer (d), endometrial cancer (e), ovarian cancer (f), colorectal cancer (g)

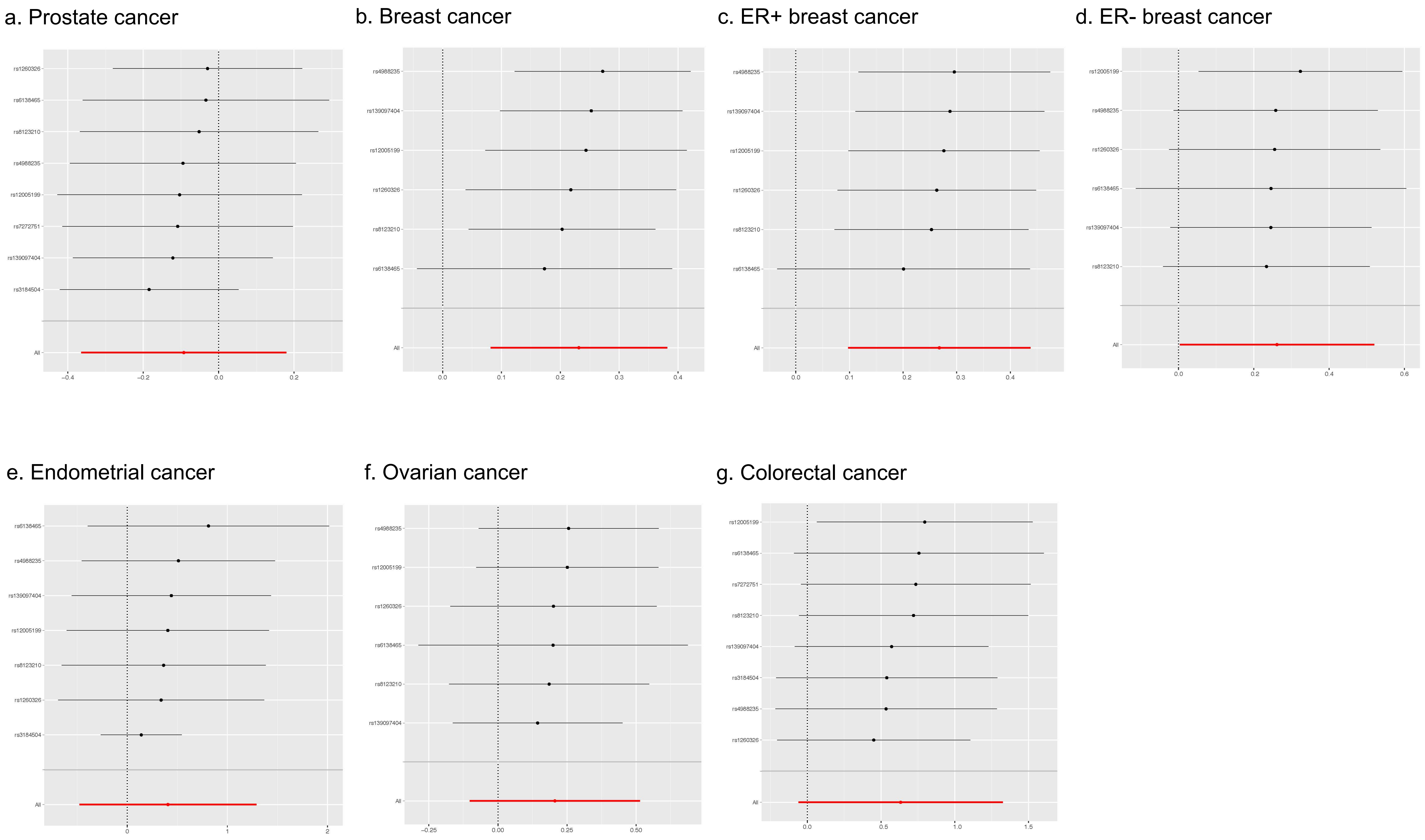

Figure S11: Leave−one−out sensitivity analysis on the association of genetically predicted acetate with prostate cancer (a), breast cancer (b), ER+ breast cancer (c), ER− breast cancer (d), endometrial cancer (e), ovarian cancer (f), colorectal cancer (g)

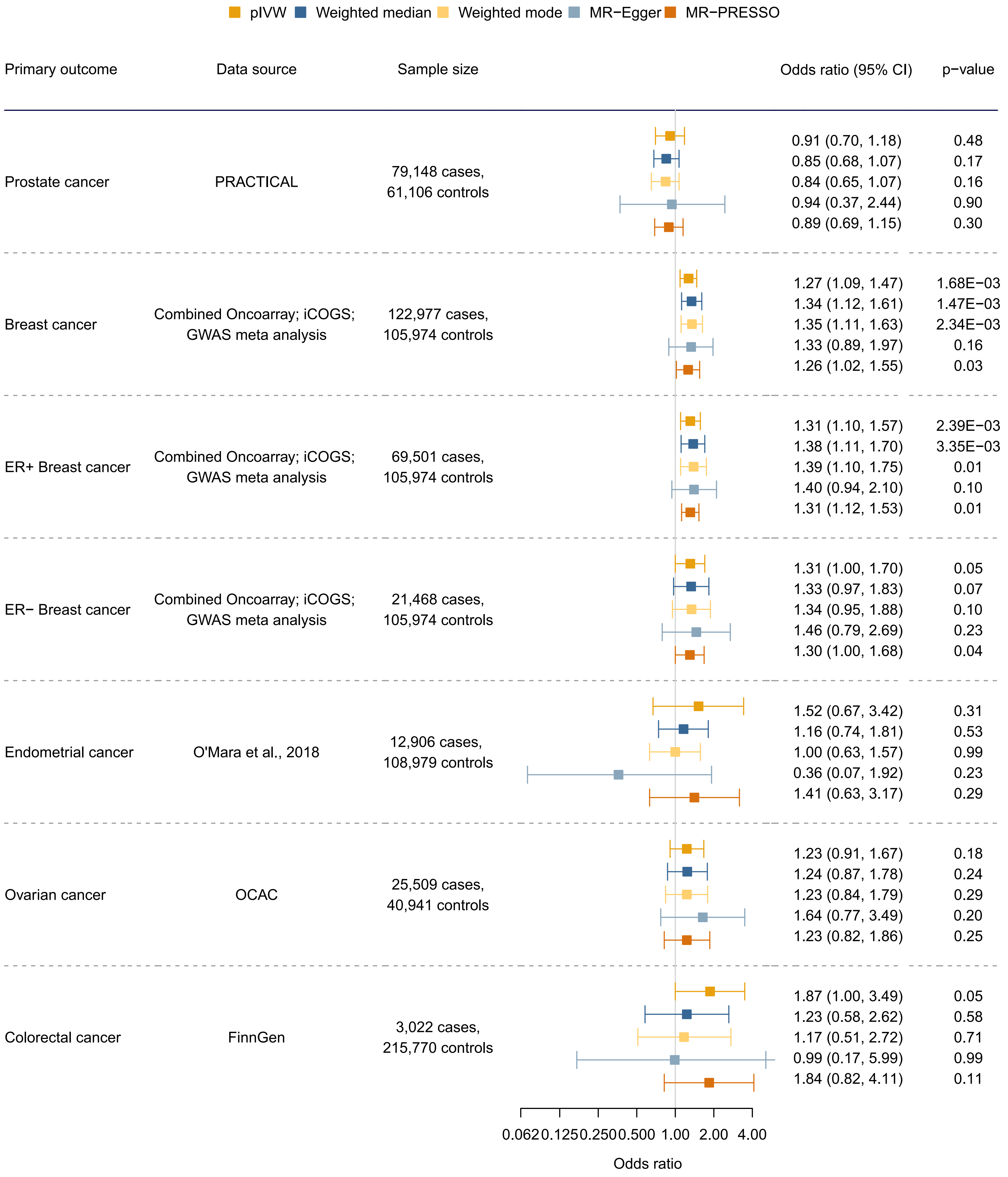

Figure S12: Sensitivity analysis on the association of genetically predicted acetate with sex hormone-related cancers using different analytic methods

Primary outcome	Data source	Sample size	Odds ratio (95% CI)	p−value
Prostate cancer	PRACTICAL	79,148 cases, 61,106 controls	0.89 (0.72, 1.09)	0.24
			0.88 (0.73, 1.07)	0.20
			0.83 (0.66, 1.05)	0.12
			0.82 (0.64, 1.06)	0.13
			0.81 (0.39, 1.70)	0.58
			0.89 (0.69, 1.13)	0.30
Breast cancer	Combined Oncoarray; iCOGS; GWAS meta analysis	122,977 cases, 105,974 controls	1.24 (1.04, 1.49)	0.02
			1.25 (1.06, 1.47)	0.01
			1.28 (1.06, 1.55)	0.01
			1.34 (1.09, 1.64)	0.01
			1.35 (0.86, 2.11)	0.19
			1.24 (0.98, 1.57)	0.08
ER− Breast cancer	Combined Oncoarray; iCOGS; GWAS meta analysis	69,501 cases, 105,974 controls	1.29 (0.98, 1.71)	0.07
			1.30 (0.98, 1.72)	0.07
			1.33 (0.96, 1.85)	0.09
			1.37 (0.96, 1.96)	0.08
			1.48 (0.80, 2.75)	0.22
			1.29 (0.96, 1.74)	0.09
ER+ Breast cancer	Combined Oncoarray; iCOGS; GWAS meta analysis	21,468 cases, 105,974 controls	1.30 (1.08, 1.56)	0.01
			1.31 (1.08, 1.58)	0.01
			1.35 (1.09, 1.68)	0.01
			1.40 (1.10, 1.78)	0.01
			1.42 (0.94, 2.13)	0.10
			1.30 (1.09, 1.56)	0.02
Endometrial cancer	O'Mara et al., 2018	12,906 cases, 108,979 controls	1.57 (0.56, 4.42)	0.39
			1.58 (0.61, 4.06)	0.34
			1.10 (0.68, 1.80)	0.69
			0.98 (0.61, 1.60)	0.95
			0.36 (0.05, 2.34)	0.28
			1.16 (0.47, 2.90)	0.68
Ovarian cancer	OCAC	25,509 cases, 40,941 controls	1.22 (0.84, 1.78)	0.29
			1.23 (0.88, 1.72)	0.23
			1.22 (0.83, 1.78)	0.31
			1.18 (0.79, 1.75)	0.42
			1.68 (0.71, 4.00)	0.24
			1.22 (0.75, 2.00)	0.35
Colorectal cancer	FinnGen	3,022 cases, 215,770 controls	1.38 (0.73, 2.60)	0.32
			1.39 (0.74, 2.62)	0.31
			1.12 (0.53, 2.39)	0.77
			1.12 (0.48, 2.61)	0.80
			1.51 (0.24, 9.52)	0.66
			1.38 (0.64, 2.96)	0.36

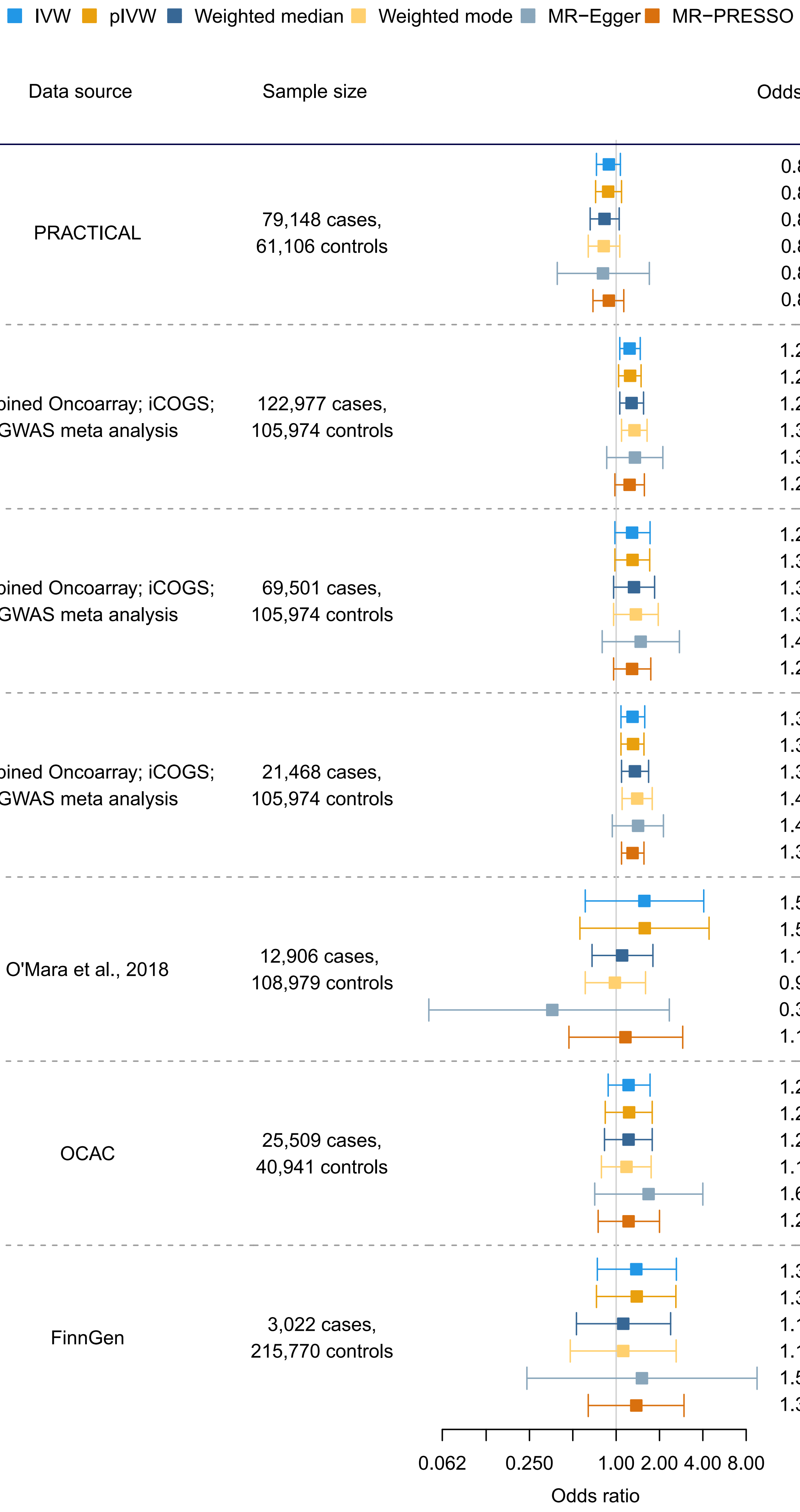

Figure S13: Sensitivity analysis on the association of genetically predicted acetate with sex hormone-related cancers using different analytic methods (removing rs1260326 and rs3184504)

Note: For endometrial cancer outcome, only SNP rs126326 was removed.

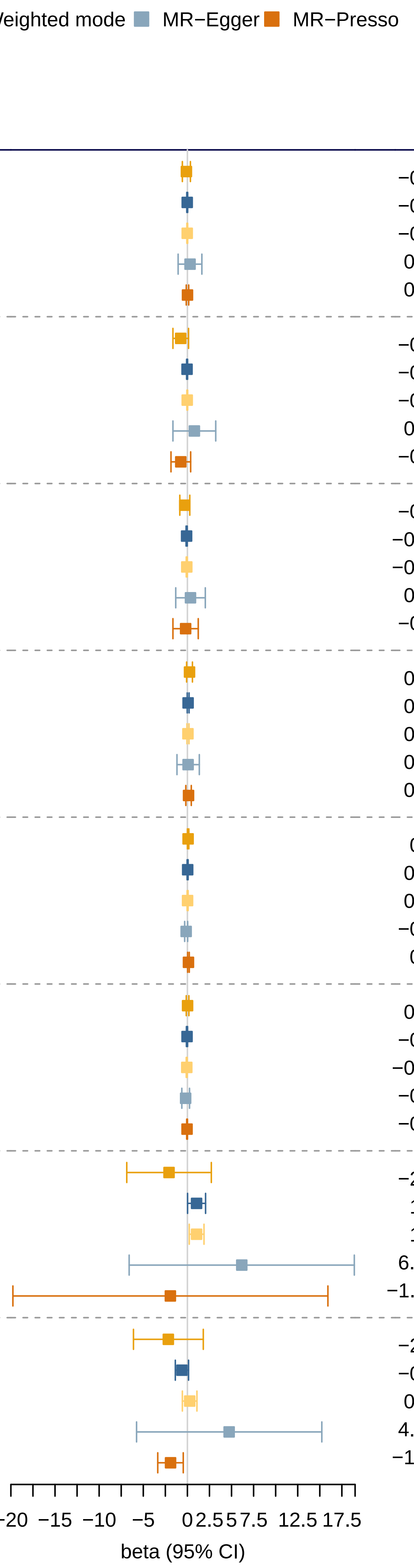

Secondary outcome	Data source	Sample size	beta (95% CI)	p−value
LDL cholesterol, SD	GLGC	Up to 842,544	−0.11 (−0.57, 0.34)	0.62
			−0.02 (−0.07, 0.03)	0.47
			−0.02 (−0.06, 0.03)	0.40
			0.30 (−1.05, 1.64)	0.66
			0.01 (−0.13, 0.15)	0.80
Triglycerides, SD	GLGC	Up to 863,369	−0.76 (−1.64, 0.13)	0.09
			−0.04 (−0.09, 0.02)	0.22
			−0.02 (−0.06, 0.03)	0.42
			0.79 (−1.64, 3.21)	0.53
			−0.75 (−1.86, 0.37)	0.16
Apolipoprotein B	UK Biobank	440,546	−0.30 (−0.86, 0.27)	0.30
			−0.09 (−0.15, −0.02)	0.01
			−0.07 (−0.13, −0.01)	0.03
			0.35 (−1.32, 2.03)	0.68
			−0.20 (−1.64, 1.23)	0.32
Fasting glucose, mmol/L	MAGIC	140,595	0.24 (−0.09, 0.57)	0.16
			0.08 (−0.02, 0.19)	0.12
			0.06 (−0.05, 0.18)	0.29
			0.08 (−1.18, 1.35)	0.90
			0.13 (−0.18, 0.44)	0.21
HbA_{1c}, SD	MAGIC	145,579	0.08 (0.00, 0.16)	0.05
			0.03 (−0.03, 0.08)	0.33
			0.02 (−0.03, 0.08)	0.40
			−0.14 (−0.31, 0.03)	0.10
			0.12 (0.03, 0.21)	0.02
Body mass index, SD	UK Biobank	461,460	0.02 (−0.13, 0.17)	0.81
			−0.04 (−0.11, 0.02)	0.19
			−0.07 (−0.13, −0.02)	0.01
			−0.20 (−0.63, 0.24)	0.38
			−0.04 (−0.08, 0.01)	0.08
Systolic blood pressure, mmHg	Combined UKB and ICBP GWAS	757,601	−2.08 (−6.87, 2.71)	0.39
			1.04 (0.02, 2.06)	0.05
			1.04 (0.21, 1.88)	0.01
			6.16 (−6.60, 18.91)	0.34
			−1.93(−19.77, 15.92)	0.40
Diastolic blood pressure, mmHg	Combined UKB and ICBP GWAS	757,601	−2.16 (−6.11, 1.80)	0.29
			−0.62 (−1.37, 0.13)	0.11
			0.26 (−0.57, 1.08)	0.54
			4.73 (−5.76, 15.23)	0.38
			−1.91 (−3.36, −0.47)	0.02

Figure S14: Sensitivity analysis on the overall association of genetically predicted acetate with common risk factors using different analytic methods

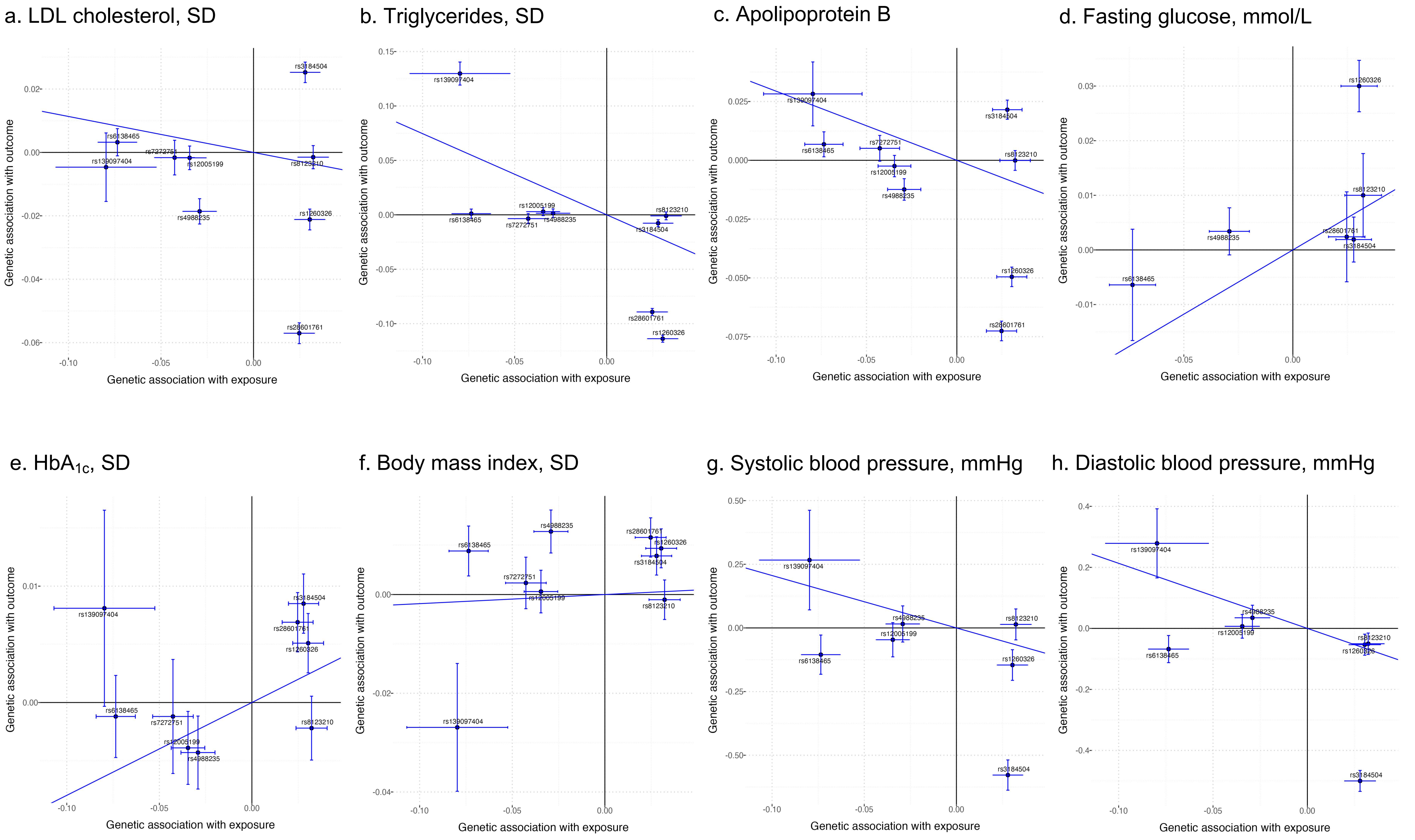

Figure S15: Scatter plot showing the overall association of each SNP with acetate and with LDL cholesterol (a), triglycerides (b), apolipoprotein B (c), fasting glucose (d), HbA_{1c} (e), body mass index (f), systolic blood pressure (g), diastolic blood pressure (h)

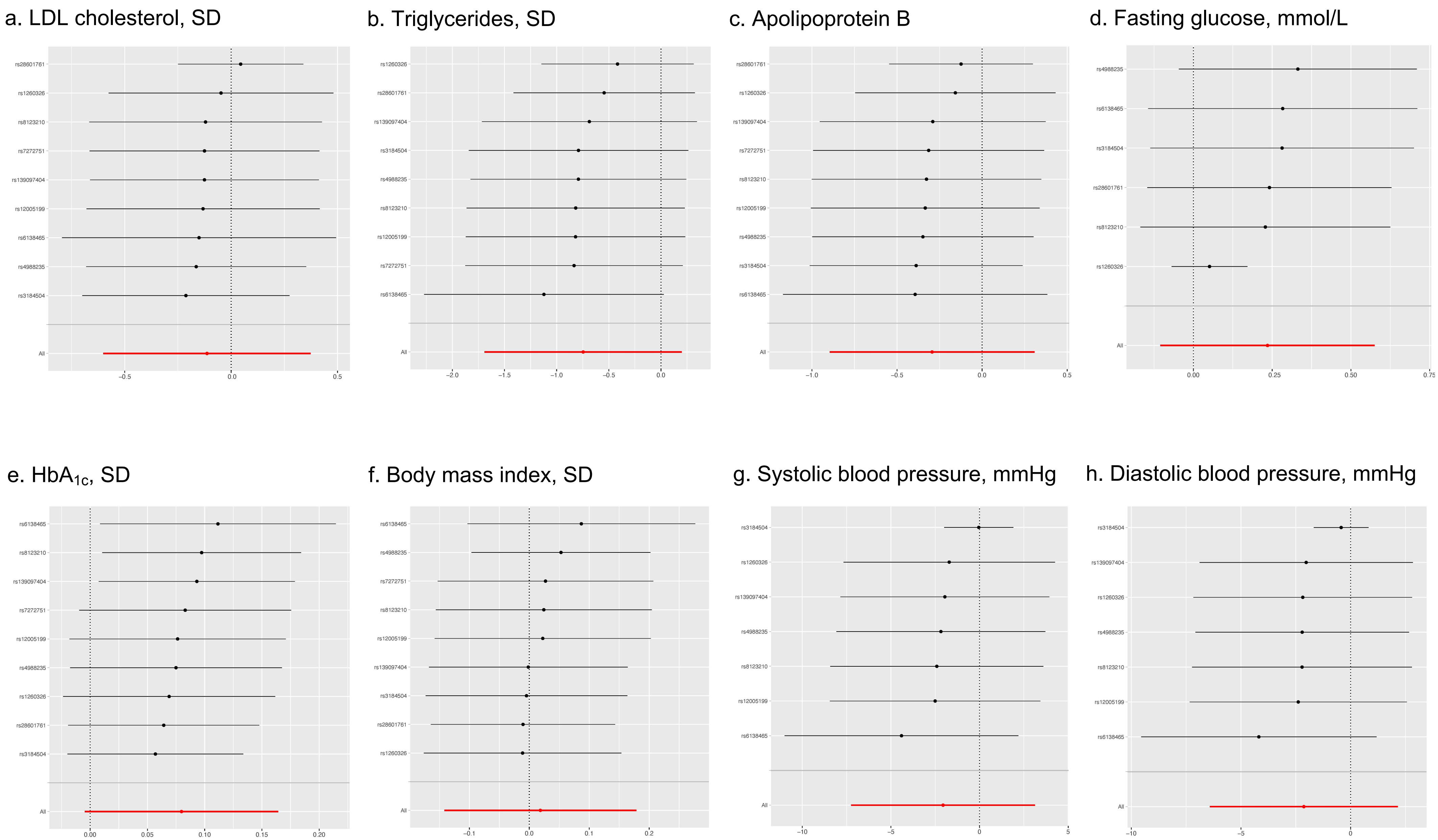

Figure S16: Leave−one−out sensitivity analysis on the overall association of genetically predicted acetate with LDL cholesterol (a), triglycerides (b), apolipoprotein B (c), fasting glucose (d), HbA_{1c} (e), body mass index (f), systolic blood pressure (g), diastolic blood pressure (h)

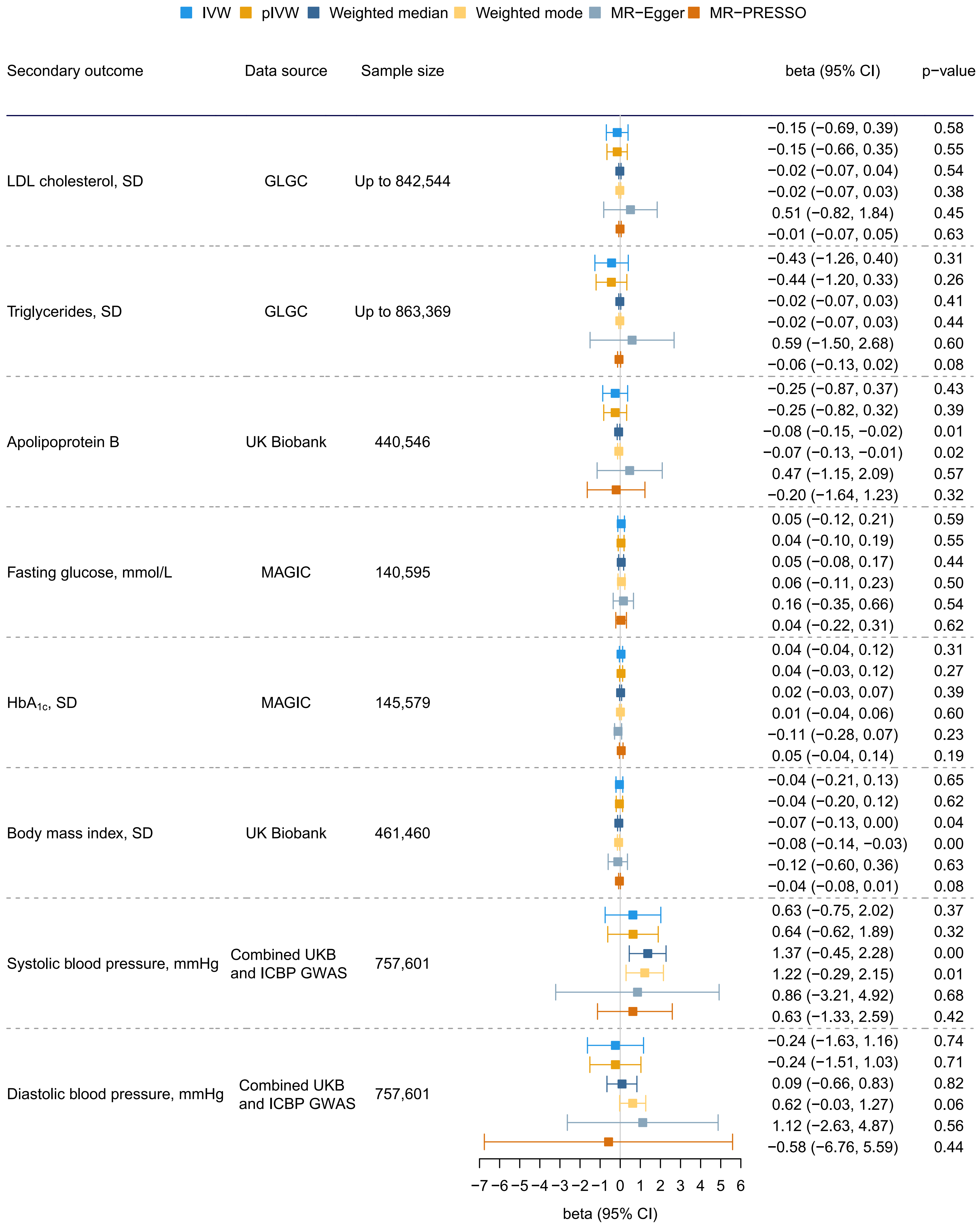

Figure S17: Sensitivity analysis on the overall association of genetically predicted acetate with common risk factors using different analytic methods (removing rs126326 and rs318454)

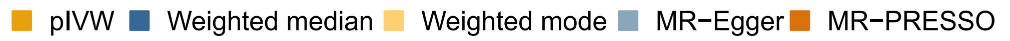

Secondary outcome	Data source	Sample size	beta (95% CI)	p−value
LDL cholesterol, SD	GLGC	Up to 686,787	−0.17 (−0.68, 0.34)	0.51
			−0.03 (−0.09, 0.02)	0.27
			−0.02 (−0.07, 0.03)	0.43
			0.27 (−1.48, 2.02)	0.76
			−0.02 (−0.10, 0.06)	0.47
Triglycerides, SD	GLGC	Up to 686,787	−0.70 (−1.59, 0.19)	0.12
			−0.04 (−0.09, 0.01)	0.13
			−0.04 (−0.08, 0.01)	0.09
			1.40 (−1.27, 4.07)	0.30
			−0.06 (0.23, 0.11)	0.28
Apolipoprotein B	UK Biobank	167,020	−0.25 (−0.83, 0.33)	0.40
			0.00 (−0.11, 0.10)	0.95
			0.03 (−0.06, 0.12)	0.47
			0.59 (−1.38, 2.57)	0.56
			−0.16 (−3.08, 2.76)	0.62
Glucose, mmol/L	UK Biobank	167,020	0.06 (−0.23, 0.34)	0.69
			−0.05 (−0.17, 0.08)	0.48
			−0.08 (−0.23, 0.06)	0.26
			−0.62 (−1.50, 0.25)	0.16
			−0.05 (0.01, 0.04)	0.19
HbA_{1c}, SD	UK Biobank	167,020	0.25 (−0.08, 0.58)	0.14
			0.15 (0.03, 0.28)	0.02
			0.00 (−0.11, 0.10)	0.97
			−0.68 (−1.64, 0.27)	0.16
			0.18 (0.05, 0.31)	0.03
Body mass index, SD	UK Biobank	167,020	0.11 (−0.09, 0.32)	0.28
			−0.03 (−0.14, 0.07)	0.57
			0.00 (−0.09, 0.09)	0.97
			−0.13 (−0.85, 0.58)	0.71
			0.06 (−0.19, 0.31)	0.51
Systolic blood pressure, mmHg	UK Biobank	167,020	−0.03 (−0.16, 0.10)	0.65
			0.02 (−0.08, 0.11)	0.70
			0.02 (−0.09, 0.13)	0.72
			0.13 (−0.32, 0.58)	0.57
			−0.01 (−0.13, 0.11)	0.89
Diastolic blood pressure, mmHg	UK Biobank	167,020	−0.11 (−0.33, 0.10)	0.29
			−0.02 (−0.11, 0.07)	0.71
			−0.01 (−0.10, 0.08)	0.84
			0.07 (−0.68, 0.82)	0.85
			−0.03 (−0.13, 0.07)	0.48

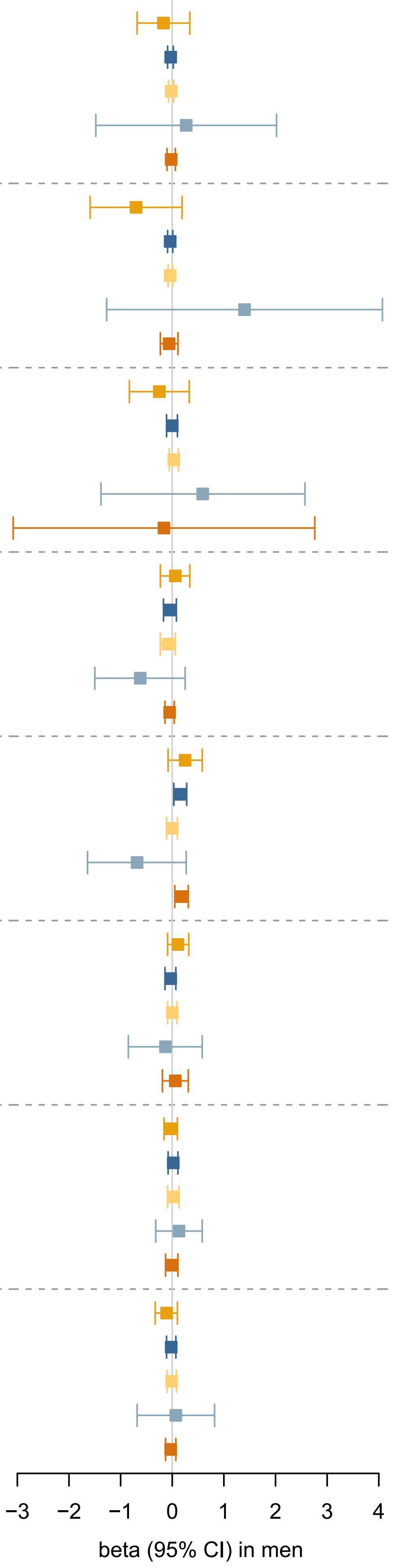

Secondary outcome	Data source	Sample size	beta (95% CI)	p−value
LDL cholesterol, SD	GLGC	Up to 570,542	−0.20 (−0.69, 0.29)	0.42
			−0.07 (−0.13, −0.02)	0.01
			−0.07 (−0.12, −0.02)	0.01
			−0.39 (−0.78, 0.00)	0.05
			−0.05 (−0.13, 0.03)	0.23
Triglycerides, SD	GLGC	Up to 570,542	−0.73 (−1.56, 0.09)	0.08
			−0.09 (−0.16, −0.03)	0.01
			−0.04 (−0.09, 0.01)	0.08
			0.10 (−0.14, 0.34)	0.44
			−0.26 (−0.78, 0.26)	0.17
Apolipoprotein B	UK Biobank	194,174	−0.27 (−0.79, 0.25)	0.31
			−0.09 (−0.18, −0.01)	0.04
			−0.07 (−0.15, 0.01)	0.07
			−0.36 (−0.66, −0.06)	0.02
			−0.06 (−0.36, 0.24)	0.47
Glucose, mmol/L	UK Biobank	194,174	0.14 (−0.03, 0.31)	0.10
			0.04 (−0.09, 0.16)	0.56
			−0.05 (−0.16, 0.06)	0.39
			−0.16 (−0.42, 0.10)	0.22
			0.17 (0.02, 0.32)	0.02
HbA_{1c}, SD	UK Biobank	194,174	0.12 (−0.13, 0.37)	0.35
			0.02 (−0.07, 0.12)	0.60
			−0.02 (−0.10, 0.07)	0.71
			−0.31 (−1.04, 0.43)	0.41
			0.10 (−0.03, 0.23)	0.04
Body mass index, SD	UK Biobank	194,174	−0.06 (−0.20, 0.08)	0.40
			−0.11 (−0.20, −0.03)	0.01
			−0.11 (−0.19, −0.03)	0.01
			−0.26 (−0.60, 0.08)	0.13
			−0.08 (−0.21, 0.05)	0.19
Systolic blood pressure, mmHg	UK Biobank	194,174	−0.09 (−0.26, 0.08)	0.30
			0.01 (−0.07, 0.10)	0.77
			0.04 (−0.04, 0.11)	0.38
			0.32 (−0.14, 0.78)	0.17
			−0.11 (−0.50, 0.28)	0.26
Diastolic blood pressure, mmHg	UK Biobank	194,174	−0.12 (−0.38, 0.15)	0.38
			−0.01 (−0.10, 0.08)	0.81
			0.02 (−0.07, 0.11)	0.62
			0.53 (−0.19, 1.26)	0.15
			−0.14 (−0.29, 0.01)	0.06

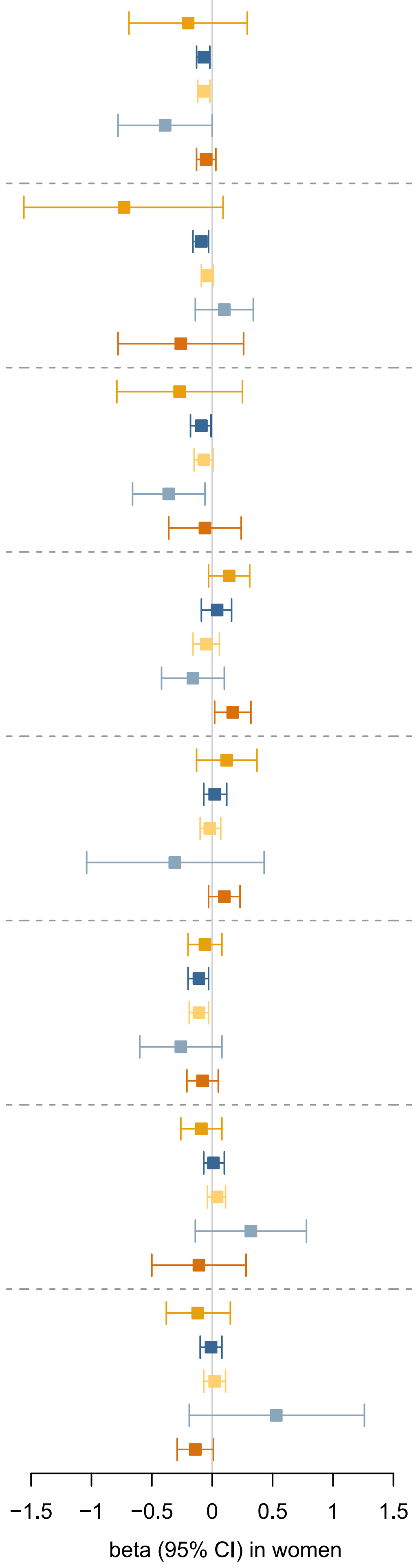

Figure S18: Sensitivity analysis on the sex-specific association of genetically predicted acetate with common risk factors by sex using different analytic methods

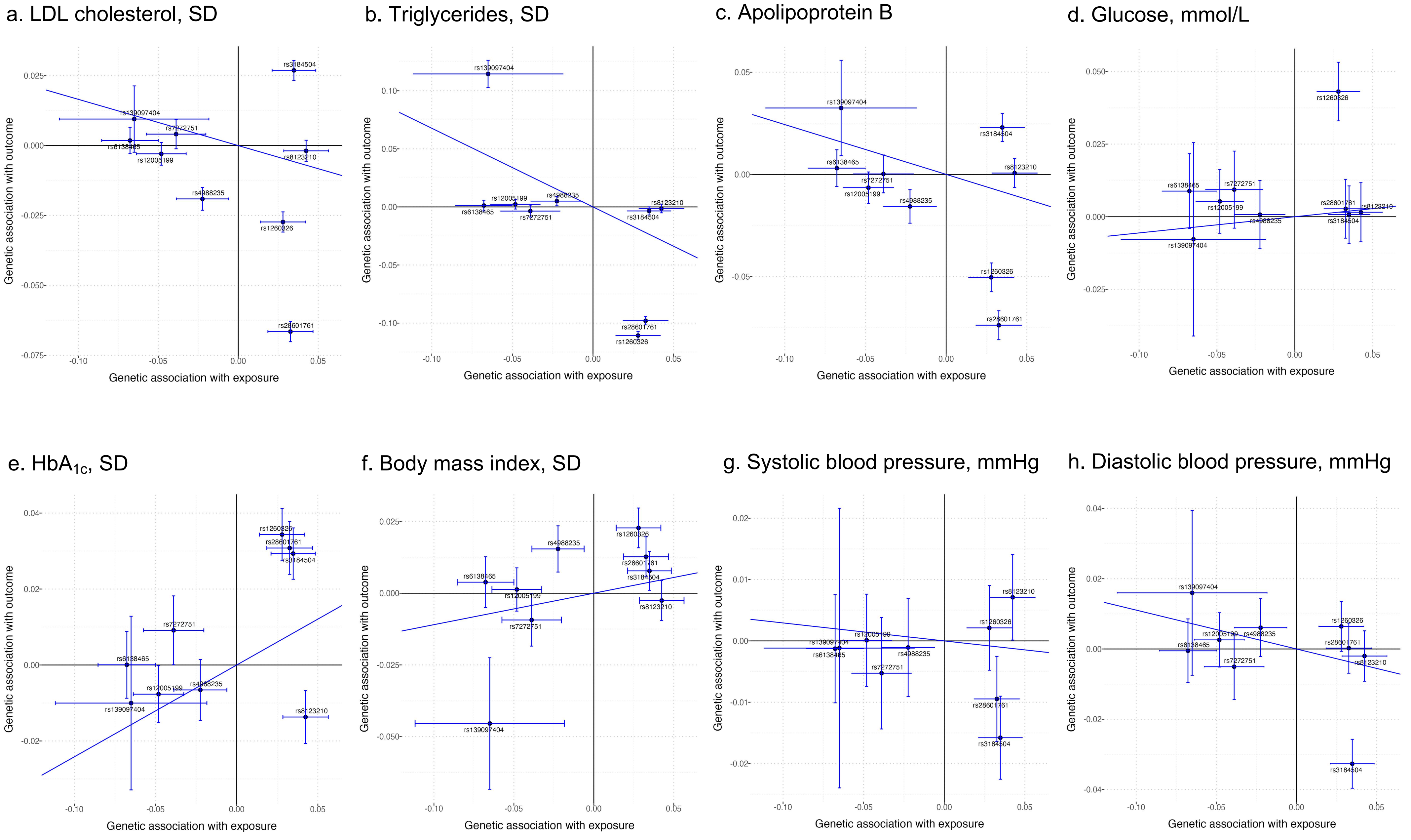

Figure S19: Scatter plot showing the sex-specific association of each SNP with acetate and with LDL cholesterol (a), triglycerides (b), apolipoprotein B (c), fasting glucose (d), HbA_{1c} (e), body mass index (f), systolic blood pressure (g), diastolic blood pressure (h) in men

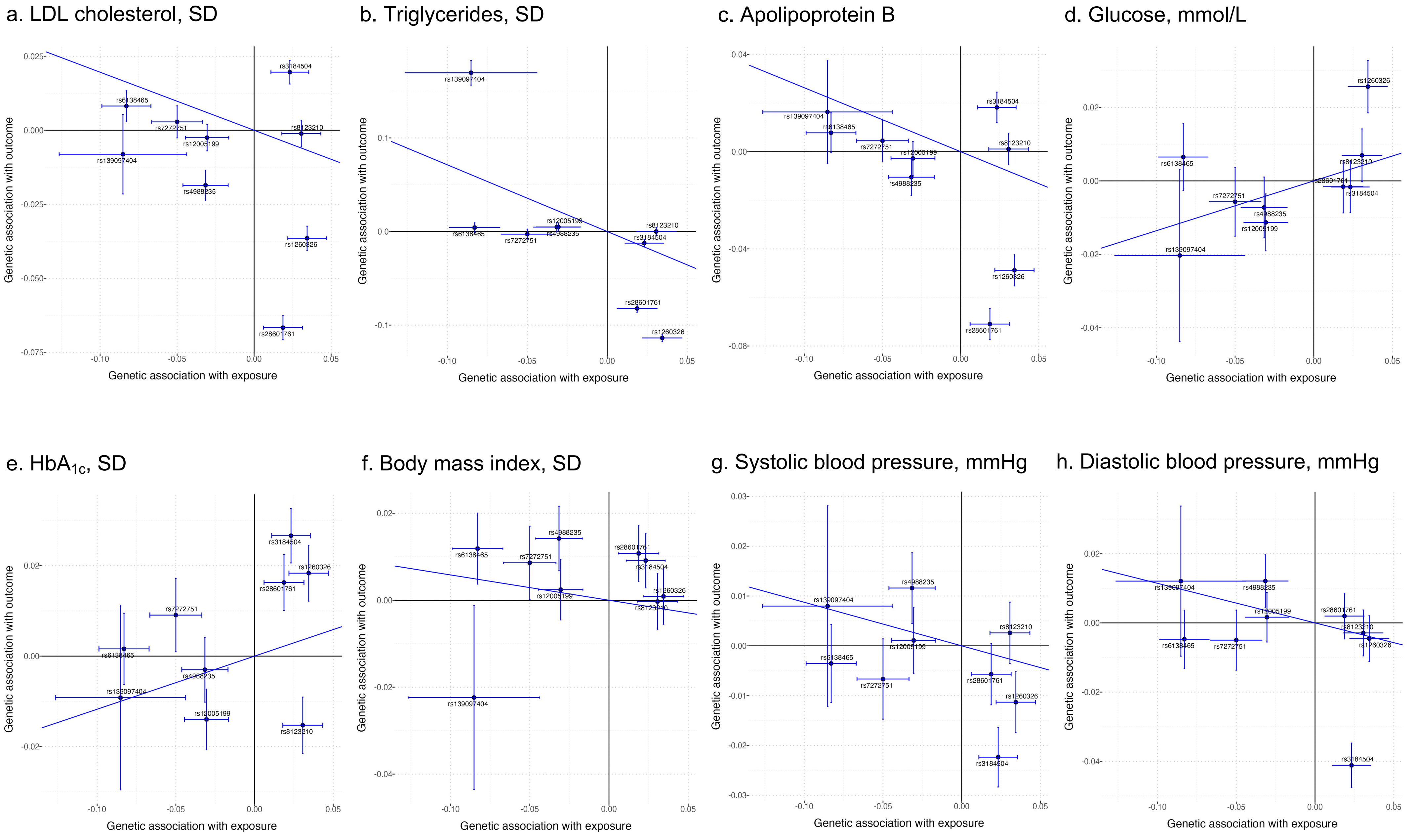

Figure S20: Scatter plot showing the sex-specific association of each SNP with acetate and with LDL cholesterol (a), triglycerides (b), apolipoprotein B (c), fasting glucose (d), HbA$_{1c}$ (e), body mass index (f), systolic blood pressure (g), diastolic blood pressure (h) in women

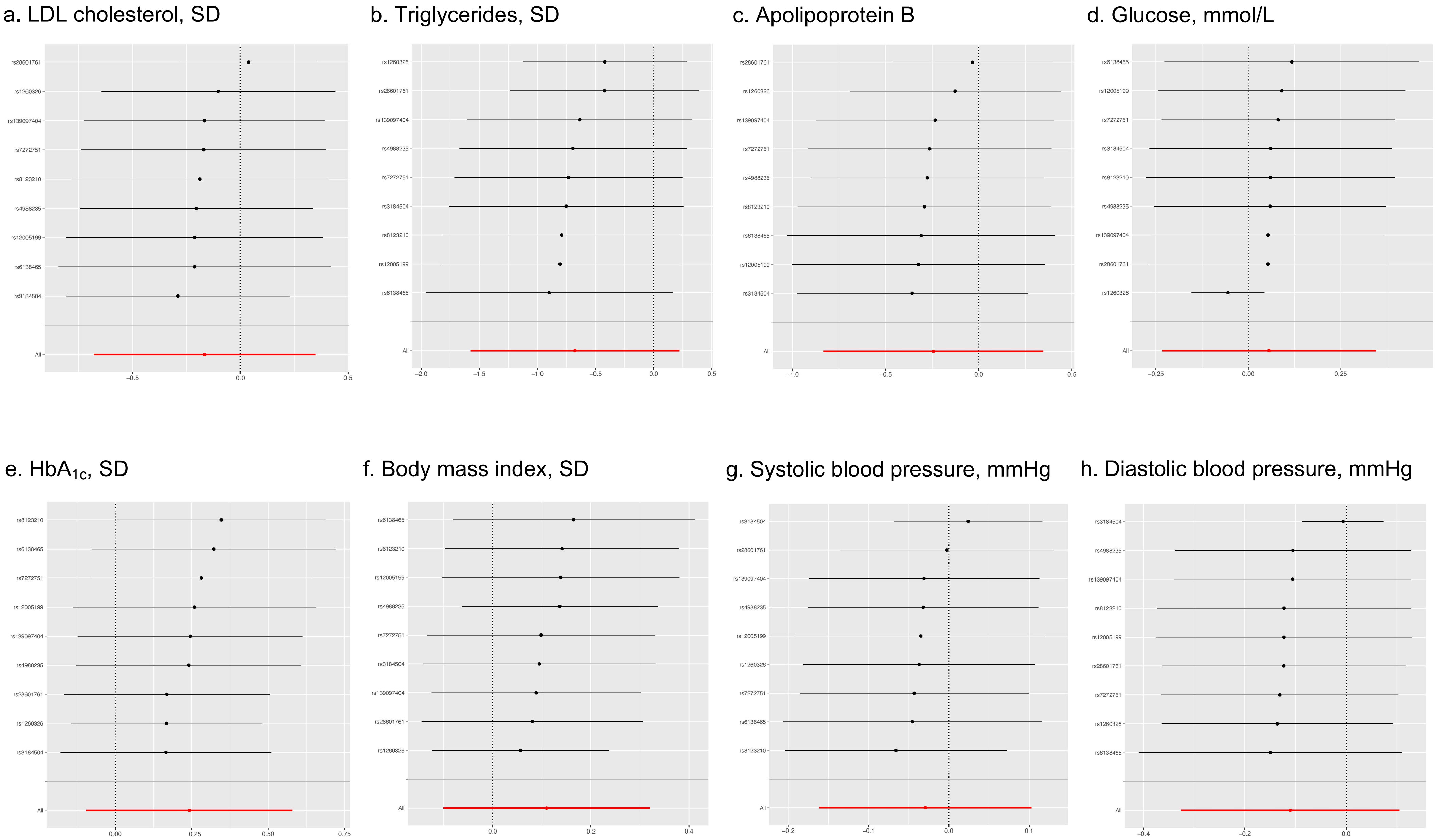

Figure S21: Leave−one−out sensitivity analysis on the sex-specific association of genetically predicted acetate with LDL cholesterol (a), triglycerides (b), apolipoprotein B (c), fasting glucose (d), HbA$_{1c}$ (e), body mass index (f), systolic blood pressure (g), diastolic blood pressure (h) in men

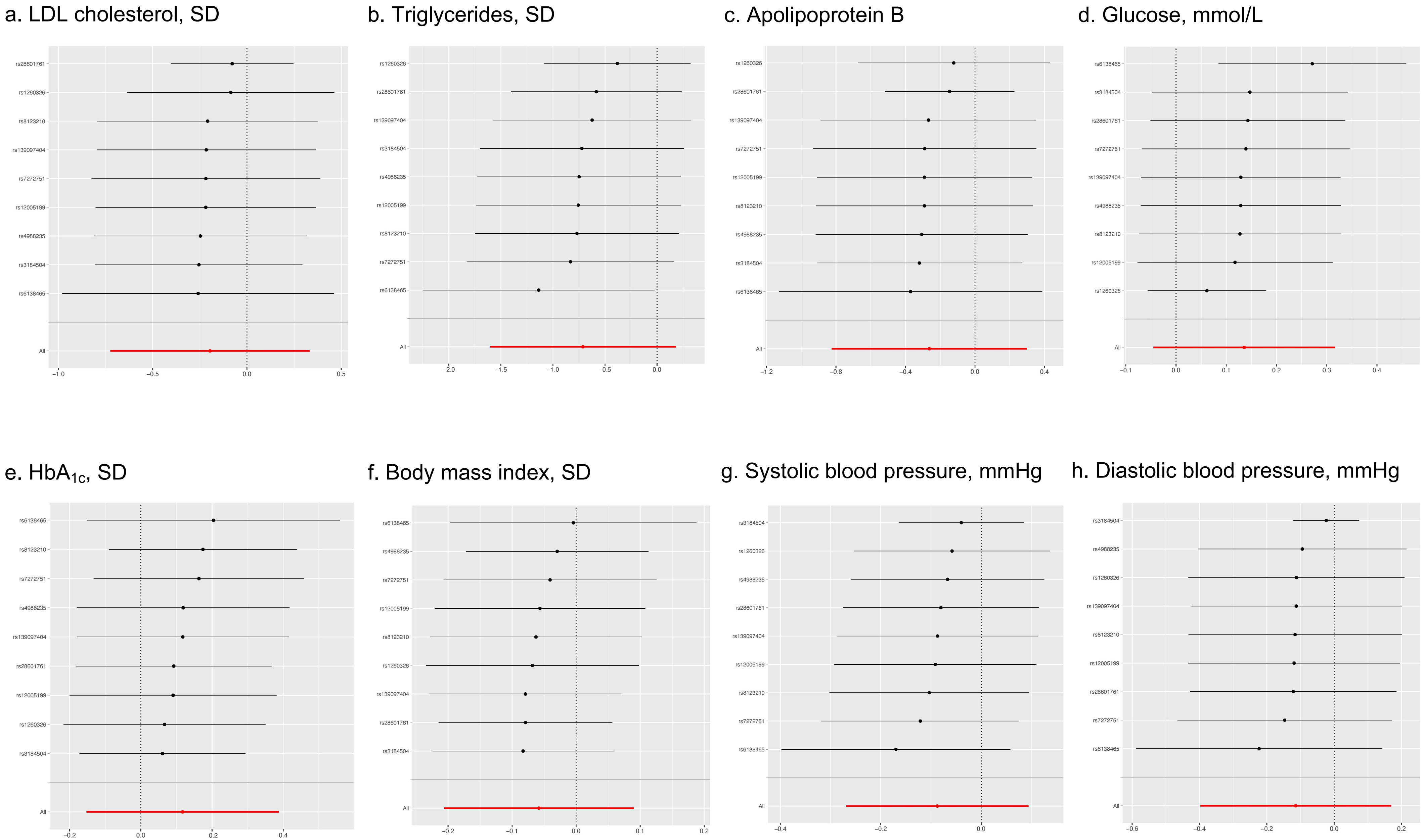

Figure S22: Leave−one−out sensitivity analysis on the sex-specific association of genetically predicted acetate with LDL cholesterol (a), triglycerides (b), apolipoprotein B (c), fasting glucose (d), HbA_{1c} (e), body mass index (f), systolic blood pressure (g), diastolic blood pressure (h) in women
