# Supplementary material for: Using Genetics to Assess the Role of Acetate in Ischemic Heart Disease, Diabetes, and Sex-Hormone-Related Cancers: A Mendelian Randomization Study
Source: Nutrients. 2024 Oct 29;16(21):3674. doi: 10.3390/nu16213674 (PMC11547320; doi:10.3390/nu16213674)
Supplement: Supplementary file 1 [file nutrients-16-03674-s001.zip › Supplemental Tables.pdf]

Table S1: Genetic variants used for acetate

SNP	effect_allele	other_allele	chr	position	function	beta	se	p-value	F-statistics	eQTL (Yes or No)*
rs1260326	C	T	2	27730940	missense	0.03	0.004	8.70E-13	51.1	Yes
rs4988235	A	G	2	136608646	intron	-0.03	0.005	5.40E-10	38.5	Yes
rs28601761	G	C	8	126500031	intron	0.02	0.004	6.80E-09	33.6	No
rs12005199	A	G	9	4763491	intergenic	-0.03	0.005	8.90E-14	55.6	No
rs3184504	C	T	12	111884608	missense	0.03	0.004	2.00E-11	44.9	Yes
rs139097404	C	T	15	43933941	intron	-0.08	0.014	1.00E-08	32.8	Yes
rs7272751	T	C	20	26002648	intron	-0.04	0.006	2.60E-14	58.0	Not available in GTEx
rs8123210	C	G	20	33486047	intron	0.03	0.004	5.70E-14	56.5	Yes
rs6138465	C	T	20	24986295	downstream	-0.07	0.005	3.80E-42	185.0	Yes

*Details were shown in Table S2

Table S2: **Detailed information on eQTL for the genetic instruments**

SNP	Relevance gene for eQTL	Tissue
rs1260326	*AC074117.10, ATRAID, C2orf16, FNDC4, GCKR, GPN1, KRTCAP3, NRBP1, PPM1G, SNX17*	Adipose- Subcutaneous, Adipose -Visceral (Omentum), Adrenal Gland, Artery- Tibial, Brain - Caudate (basal ganglia), Brain - Cerebellar Hemisphere, Brain -Cerebellum, Breast - Mammary Tissue, Cells - Cultured fibroblasts, Colon-Transverse, Esophagus - Gastroesophageal Junction, Esophagus – Mucosa, Esophagus-Muscularis, Heart - Atrial Appendage, Heart-Left Ventricle, Lung, Muscle-Skeletal, Nerve -Tibial, Pancreas, Prostate, Skin, Small Intestine - Terminal Ileum, Spleen, Testis, Thyroid, Whole Blood
rs4988235	*CCNT2, DARS, DARS-AS1, MAP3K19, MCM6, UBXN4*	Adipose- Subcutaneous, Adipose- Visceral (Omentum), Artery - Aorta, Artery-Coronary, Artery - Tibial, Brain - Cerebellar Hemisphere, Brain - Cerebellum, Cells - Cultured fibroblasts, Colon - Sigmoid, Colon - Transverse, Esophagus -Gastroesophageal Junction, Esophagus -Mucosa, Esophagus -Muscularis, Heart - Atrial Appendage, Heart - Left Ventricle, Lung, Nerve- Tibial, Pancreas, Skin, Spleen, Testis, Thyroid, Whole Blood
rs3184504	*ADAM1B, ALDH2, LINC01405, MAPKAPK5, TMEM116*	Adipose- Subcutaneous, Artery - Aorta, Artery- Tibial, Brain - Nucleus accumbens (basal ganglia), Colon – Sigmoid, Esophagus – Mucosa, Heart - Atrial Appendage, Muscle – Skeletal, Skin
rs139097404	*AC011330.5, ADAL, CATSPER2P1, CDAN1, CKMT1A, E LL3, HYPK, LCMT2, PPIP5K1, STRC, STRCP1, TP53BP 1, TUBGCP4*	Adipose – Subcutaneous, Adipose - Visceral (Omentum), Adrenal Gland, Artery – Aorta, Artery – Coronary, Artery – Tibial, Brain – Amygdala, Brain - Anterior cingulate cortex (BA24), Brain - Caudate (basal ganglia), Brain -Cortex, Brain-Hypothalamus, Brain - Nucleus accumbens (basal ganglia), Brain - Putamen (basal ganglia), Breast - Mammary Tissue, Cells - Cultured fibroblasts, Colon - Sigmoid, Colon - Transverse, Esophagus - Gastroesophageal Junction, Esophagus - Mucosa, Esophagus-Muscularis, Heart - Atrial Appendage, Liver, Lung, Muscle – Skeletal, Nerve – Tibial Pancreas, Pituitary, Skin, Spleen, Stomach, Testis, Thyroid
rs8123210	*ACSS2, DYNLRB1, EDEM2, FAM83C, GGT7, GSS, ITC H, MAP1LC3A, MMP24-AS1, MYH7B, NCOA6, NFS1, PI GU, PROCR, RALY, RP5-1125A11.7, TP53INP2, TRPC4 AP, UQCC1*	Adipose- Subcutaneous, Adipose - Visceral (Omentum), Artery- Aorta, Artery- Tibial, Brai n - Anterior cingulate cortex (BA24), Brain - Caudate (basal ganglia), Brain - Cerebellar He misphere, Brain - CerebellumBrain – Cortex, Brain - Frontal Cortex (BA9), Brain - Nucleus accumbens (basal ganglia), Cells - Cultured fibroblasts, Colon – Sigmoid, Colon - Transvers e, Esophagus - Gastroesophageal Junction, Esophagus -Mucosa,Esophagus- Muscularis, Hea rt - Atrial Appendage, Heart - Left Ventricle, Lung, Muscle - Skeletal Nerve- Tibial, Ovary, Pancreas, Pituitary Prostate, Skin, Small Intestine - Terminal Ileum, Spleen, Stomach, Testi s, Thyroid, Whole Blood
rs6138465	*ABHD12, ACSS1, APMAP, BSNDP2, CST7, AM182B, NINL, PYGB, RP4-568C11.4*	Adrenal Gland, Brain - Cerebellar Hemisphere, Brain – Cerebellum, Cells - Cultured fibroblasts, Colon – Transverse, Esophagus - Gastroesophageal Junction, Esophagus-Muscularis, Nerve-Tibial, Pancreas, Pituitary, Skin, Spleen, Testis, Thyroid, Whole Blood

Table S3: Associations of genetic variants instrumenting acetate with potential confounders

Confounders	SNP	effect_allele	other_allele	beta	p-value
Townsend index	rs3184504	C	T	-0.004	0.07
Townsend index	rs139097404	C	T	-0.008	0.29
Townsend index	rs4988235	A	G	-0.001	0.74
Townsend index	rs1260326	C	T	0.002	0.38
Townsend index	rs6138465	C	T	0.002	0.58
Townsend index	rs7272751	T	C	0.003	0.29
Townsend index	rs8123210	C	G	-0.004	0.06
Townsend index	rs28601761	G	C	0.002	0.48
Townsend index	rs12005199	A	G	-0.001	0.74
Age completed full time education	rs3184504	C	T	0.005	0.04
Age completed full time education	rs139097404	C	T	0.007	0.36
Age completed full time education	rs4988235	A	G	-0.009	0.0004
Age completed full time education	rs1260326	C	T	0.003	0.22
Age completed full time education	rs6138465	C	T	0.001	0.65
Age completed full time education	rs7272751	T	C	-0.001	0.63
Age completed full time education	rs8123210	C	G	0.005	0.05
Age completed full time education	rs28601761	G	C	0.006	0.01
Age completed full time education	rs12005199	A	G	-0.0003	0.90
Current smoking	rs3184504	C	T	0.0001	0.92
Current smoking	rs139097404	C	T	0.001	0.82
Current smoking	rs4988235	A	G	-0.0001	0.86
Current smoking	rs1260326	C	T	-0.001	0.12
Current smoking	rs6138465	C	T	0.002	0.02
Current smoking	rs7272751	T	C	0.002	0.11
Current smoking	rs8123210	C	G	-0.001	0.33
Current smoking	rs28601761	G	C	0.0000	0.99
Current smoking	rs12005199	A	G	0.0003	0.73
Alcohol intake frequency	rs3184504	C	T	-0.001	0.81
Alcohol intake frequency	rs139097404	C	T	0.004	0.76
Alcohol intake frequency	rs4988235	A	G	0.011	0.01
Alcohol intake frequency	rs1260326	C	T	-0.049	1.28E-43
Alcohol intake frequency	rs6138465	C	T	0.002	0.61
Alcohol intake frequency	rs7272751	T	C	0.001	0.81
Alcohol intake frequency	rs8123210	C	G	-0.0002	0.95
Alcohol intake frequency	rs28601761	G	C	-0.016	4.51E-06
Alcohol intake frequency	rs12005199	A	G	0.000	0.99
Time spent on moderate physical activity	rs3184504	C	T	0.009	0.09
Time spent on moderate physical activity	rs139097404	C	T	0.011	0.56
Time spent on moderate physical activity	rs4988235	A	G	-0.014	0.03
Time spent on moderate physical activity	rs1260326	C	T	-0.003	0.59
Time spent on moderate physical activity	rs6138465	C	T	0.010	0.17
Time spent on moderate physical activity	rs7272751	T	C	0.001	0.86
Time spent on moderate physical activity	rs8123210	C	G	0.001	0.88
Time spent on moderate physical activity	rs28601761	G	C	0.0002	0.97
Time spent on moderate physical activity	rs12005199	A	G	-0.003	0.59
Time spent on vigorous physical activity	rs3184504	C	T	0.007	0.14

Time spent on vigorous physical activity	rs139097404	C	T	0.013	0.41
Time spent on vigorous physical activity	rs4988235	A	G	-0.004	0.50
Time spent on vigorous physical activity	rs1260326	C	T	0.003	0.54
Time spent on vigorous physical activity	rs6138465	C	T	0.005	0.40
Time spent on vigorous physical activity	rs7272751	T	C	0.010	0.11
Time spent on vigorous physical activity	rs8123210	C	G	-0.001	0.77
Time spent on vigorous physical activity	rs28601761	G	C	0.003	0.56
Time spent on vigorous physical activity	rs12005199	A	G	0.0002	0.97

Table S4: Heterogeneity statistics for overall and sex-specific analyses

Outcome, sex, data source (if needed)	Cochran's Q statistic	Heterogeneity p value
Primary outcomes		
Overall		
IHD, CARDIoGRAMplusC4D	69.0	1.7E-13
IHD, FinnGen	42.6	1.4E-07
Type 2 diabetes, Diagram	122.4	2.4E-23
Type 2 diabetes, FinnGen	34.2	6.1E-06
Colorectal cancer	9.5	2.2E-01
Sex-specific		
IHD, men	80.8	9.5E-15
IHD, women	28.6	1.7E-04
Type 2 diabetes, men	43.0	3.4E-07
Type 2 diabetes, women	21.1	3.6E-03
Prostate cancer, men	19.0	8.2E-03
Breast cancer, women	5.6	3.5E-01
ER+ breast cancer, women	2.2	0.8
ER- breast cancer, women	2.6	0.8
Endometrial cancer, women	43.4	9.7E-08
Ovarian cancer, women	5.3	0.4
Secondary outcomes		
Overall		
LDL cholesterol, overall	1602.0	<1.0E-300
Triglycerides, overall	6400.6	<1.0E-300
Apolipoprotein B, overall	1681.5	2.2E-03
Fasting glucose, overall	122.7	8.3E-25
HbA_{1c}, overall	74.4	6.7E-13
BMI, overall	131.5	1.4E-24
SBP, overall	365.9	6.0E-76
DBP, overall	749.3	1.4E-158
Sex-specific		
LDL cholesterol, men	1742.1	<1.0E-300
LDL cholesterol, women	1406.3	2.4E-298
Triglycerides, men	5449.3	<1.0E-300
Triglycerides, women	4132.2	<1.0E-300
Apolipoprotein B, men	629.9	8.8E-131
Apolipoprotein B, women	664.0	3.9E-138
Fasting glucose, men	74.0	8.0E-13
Fasting glucose, women	55.8	3.1E-09
HbA_{1c}, men	216.5	2.1E-42
HbA_{1c}, women	166.4	7.4E-32
BMI, men	83.0	1.2E-14
BMI, women	46.4	2.0E-07
SBP, men	33.5	6.0E-05

SBP, women	76.6	2.4E-13
DBP, men	82.9	1.3E-14
DBP, women	160.8	1.1E-30

Table S5: Outliers identified in MR-PRESSO analysis

Outcome, data source/sex	Outliers
Primary outcomes	
Overall	
IHD, CARDIoGRAMplusC4D	rs3184504, rs6138465
IHD, FinnGen	rs3184504, rs8123210
Type 2 diabetes, Diagram	rs3184504, rs139097404, rs1260326
Type 2 diabetes, FinnGen	rs1260326, rs3184504
Sex-specific	
IHD, men	rs3184504, rs6138465, rs7272751, rs28601761
IHD, women	rs3184504
Type 2 diabetes, men	rs1260326
Type 2 diabetes, women	rs1260326, rs6138465
Prostate cancer, men	rs1260326, rs3184504
Endometrial cancer, women	rs3184504, rs6138465
Secondary outcomes	
Overall	
LDL cholesterol	rs12005199, rs1260326, rs28601761, rs3184504, rs4988235, rs6138465
Apolipoprotein B	rs12005199, rs1260326, rs28601761, rs3184504, rs4988235, rs6138465, rs8123210
Fasting glucose	rs1260326, rs4988235, rs6138465
HbA_{1c}	rs139097404, rs28601761, rs3184504, rs6138465, rs8123210
BMI	rs1260326, rs139097404, rs28601761, rs3184504, rs4988235, rs6138465
SBP	rs12005199, rs1260326, rs3184504, rs6138465, rs8123210
DBP	rs12005199, rs3184504, rs6138465
Sex-specific	
LDL cholesterol, men	rs4988235, rs1260326, rs3184504, rs28601761
LDL cholesterol, women	rs28601761, rs3184504, rs4988235, rs1260326
Triglycerides, men	rs139097404, rs6138465, rs7272751, rs1260326, rs3184504, rs28601761
Triglycerides, women	rs139097404, rs28601761, rs6138465, rs7272751, rs8123210, rs1260326
Apolipoprotein B, men	rs12005199, rs1260326, rs28601761, rs3184504, rs4988235, rs6138465, rs8123210
Apolipoprotein B, women	rs1260326, rs28601761, rs3184504, rs4988235, rs6138465, rs8123210
Fasting glucose, men	rs1260326
Fasting glucose, women	rs1260326, rs6138465
HbA_{1c}, men	rs1260326, rs28601761, rs3184504, rs6138465, rs7272751, rs8123210
HbA_{1c}, women	rs12005199, rs1260326, rs28601761, rs3184504, rs6138465, rs7272751, rs8123210
BMI, men	rs1260326, rs139097404, rs28601761, rs4988235, rs6138465
BMI, women	rs28601761, rs3184504, rs4988235
SBP, men	rs3184504, rs8123210
SBP, women	rs1260326, rs3184504, rs6138465, rs7272751
DBP, men	rs1260326, rs3184504
DBP, women	rs3184504, rs6138465, rs7272751

Table S6: MR-Egger intercept p-value

Outcomes	MR-Egger intercept p-value
Primary outcomes	
Overall	
IHD, Cardiogram	0.20
IHD, FinnGen	0.71
Type 2 diabetes, Diagram	0.53
Type 2 diabetes, FinnGen	0.63
Colorectal cancer, FinnGen	0.47
Sex-specific	
IHD, men	0.27
IHD, women	0.21
Type 2 diabetes, men	0.19
Type 2 diabetes, women	0.90
Prostate cancer, men	0.94
Breast cancer, women	0.78
ER+ breast cancer, women	0.70
ER- breast cancer, women	0.68
Endometrial cancer, women	0.06
Ovarian cancer, women	0.41
Secondary outcomes	
Overall	
LDL cholesterol, GLGC	0.52
Triglycerides, GLGC	0.18
Apolipoprotein B, UK Biobank	0.42
Fasting glucose, MAGIC	0.81
HbA1c, MAGIC	0.01
BMI, UKB	0.30
SBP, Combined UKB and ICBP GWAS	0.17
DBP, Combined UKB and ICBP GWAS	0.17
Sex-specific	
LDL cholesterol, men	0.61
LDL cholesterol, women	0.01
Triglycerides, men	0.11
Triglycerides, women	0.11
Apolipoprotein B, men	0.38
Apolipoprotein B, women	4.46E-03
Fasting glucose, men	0.11
Fasting glucose, women	0.07
HbA1c, men	0.05

HbA1c, women	0.32
BMI, men	0.48
BMI, women	0.36
SBP, men	0.47
SBP, women	0.08
DBP, men	0.62
DBP, women	0.05

Table S7: Power calculation

Outcomes	Sex	Sample size (case/control for IHD, type 2 diabetes and cancer)	Effect size detected at the fixed sample size and at the power of 0.8 (OR for IHD, type 2 diabetes and cancer; beta coefficient for other outcomes)
IHD	Overall	547,261 (122,733/424,528)	0.88
	Men	179,904 (31,127/148,777)	0.78
	Women	212,060 (16,286/195,774)	0.72
Type 2 diabetes	Overall	898,130 (74,124/824,006)	1.16
	Men	425,613 (41,846/383,767)	1.23
	Women	464,389 (30,053/434,336)	1.27
Prostate cancer	Men	140,254 (79,148/61,106)	0.81
Breast cancer	Women	228,951 (122,977/105,974)	1.18
ER+ breast cancer	Women	175,475 (69,501/105,974)	1.21
ER- breast cancer	Women	127,442 (21,468/105,974)	1.35
Endometrial cancer	Women	121,885 (12,906/108,979)	1.45
Ovarian cancer	Women	66,450 (25,509/40,941)	1.37
Colorectal cancer	Overall	218,792 (3,022/215,770)	2.07
Fasting glucose	Overall	140,595	0.11
	Men	67,506	0.15
	Women	73,089	0.15
HbA_{1c}	Overall	145,579	0.10
	Men	167,020	0.10
	Women	194,174	0.09
LDL cholesterol	Overall	Up to 842,544	0.04
	Men	Up to 686,787	0.05
	Women	Up to 570,542	0.05
Triglycerides	Overall	Up to 863,369	0.04
	Men	Up to 686,787	0.05
	Women	Up to 570,542	0.05
Apolipoprotein B	Overall	440,546	0.06
	Men	167,020	0.10
	Women	194,174	0.09
Body mass index	Overall	461,460	0.06
	Men	167,020	0.10
	Women	194,174	0.09
SBP	Overall	757,601	0.05
	Men	167,020	0.10
	Women	194,174	0.09
DBP	Overall	757,601	0.05
	Men	167,020	0.10
	Women	194,174	0.09

The power calculations for the MR analysis were performed using the online tool available at https://sb452.shinyapps.io/power/. The effect size shown in this table was in the scale of odds ratio of IHD, type 2 diabetes and cancer, and SD of the other continuous outcomes.

Table S8: MR analysis on the associations of genetically predicted IHD and breast cancer with acetate

Exposure	Outcome	Method	beta	se	p-value
IHD	Acetate	IVW	-0.02	0.01	0.05
		pIVW	-0.02	0.01	0.04
		Weighted median	0.00	0.01	0.74
		Weighted mode	0.00	0.01	0.81
		MR-Egger	0.01	0.03	0.75
		MR-PRESSO	-0.01	0.01	0.19
Breast cancer	Acetate	IVW	0.00	0.01	0.52
		pIVW	0.00	0.01	0.52
		Weighted median	0.01	0.01	0.59
		Weighted mode	0.01	0.01	0.49
		MR-Egger	0.00	0.01	0.79
		MR-PRESSO	0.00	0.01	0.52

Table S9: MR analysis on the associations of genetically predicted acetate with fasting glucose, SBP, DBP in different GWAS

Outcome	Data source	beta	se	p-value
Fasting glucose	UK Biobank	0.11	0.12	0.36
	MAGIC	0.24	0.17	0.18
SBP	UK Biobank	-1.03	1.78	0.56
	Combined UKB and ICBP GWAS	-2.06	2.65	0.44
DBP	UK Biobank	-1.58	1.58	0.32
	Combined UKB and ICBP GWAS	-2.13	2.19	0.33
